# Evaluation of extraction methodologies for PFAS analysis in mascara: a comparative study of SPME and automated µSPE

**DOI:** 10.1007/s00216-025-05908-x

**Published:** 2025-05-24

**Authors:** Aghogho A. Olomukoro, Lucas Lüthy, Tom Flug, Emanuela Gionfriddo

**Affiliations:** 1 Department of Chemistry, University at Buffalo, The State University of New York, Buffalo, NY 14260-3000 USA; 2 CTC Analytics AG, Industriestrasse 20, 4222 Zwingen, Switzerland

**Keywords:** PFAS, Cosmetics, Solid-phase microextraction, Automated micro-solid-phase extraction

## Abstract

Research efforts have primarily focused on identifying per- and polyfluoroalkyl substances (PFAS) in common environmental media like water, air, soil, and biological samples. However, there is limited research on PFAS detection in complex samples such as personal care products, including cosmetics. PFAS are used in cosmetics for emulsification, surfactant action, and stabilization, and have been detected in products such as foundation, powders, and nail polish. The complexity of cosmetic formulations, with various additives, makes the analysis of these samples extremely challenging. This study aimed to explore and develop convenient extraction methods to accurately quantify eight anionic PFAS in mascara products. Solid-phase microextraction (SPME) and automated micro solid-phase extraction (µSPE) were evaluated, and quantification was performed using liquid chromatography-tandem mass spectrometry (LC–MS/MS). Six mascara products, including waterproof and non-waterproof types, were analyzed, optimizing methanol–water mixtures as dispersive media to maximize PFAS recovery. Elution solution composition, volume, and dispensing speed were optimized for the µSPE method to ensure quantitative elution of the PFAS from the extraction phase. For the SPME method, the extraction time was optimized to account for the varying diffusion behavior of PFAS in the mixed-phase medium. Both extraction methods were evaluated in terms of greenness and practicality, with SPME achieving the best overall scores. Method validation demonstrated good linearity (0.025 to 25 ng/g) for both protocols, with µSPE providing lower limits of quantification (LOQ) for the most hydrophobic PFAS. 6:2 diPAP was quantified in real samples at concentrations ranging from 1.26 to 3.48 ng/g in 4 of the 9 mascaras tested.

**Graphical Abstract:**

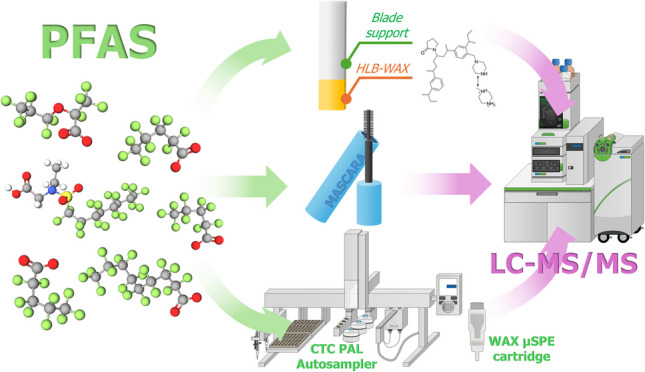

**Supplementary Information:**

The online version contains supplementary material available at 10.1007/s00216-025-05908-x.

## Introduction

Per- and polyfluoroalkyl substances (PFAS) have been widely used for decades in the production of various commercial and consumer products. PFAS widespread use stems from their unique chemical structure, which provides thermal and chemical stability, as well as their ability to repel oil and water [1, 2]. PFAS consists of an alkyl chain that can be either fully fluorinated (per-) or partially fluorinated (poly-), with a functional head group containing sulfonate, carboxylate, or phosphate groups, giving PFAS amphiphilic and surfactant properties [1, 3, 4]. In recent years, legacy PFAS like perfluorooctanesulfonic acid (PFOS) and perfluorooctanoic acid (PFOA) were phased out and replaced with alternative PFAS types, such as shorter-chain and per- or poly-fluoroether compounds, which continue to increase their environmental persistence and associated health risks [1, 5, 6]. PFAS are extensively used in products like food packaging materials, non-stick cookware, stain- and water-resistant clothing, firefighting foams, and medical equipment [3, 6–8]. However, a lesser-known application of PFAS is their use in cosmetic products [1], where they fulfill various functions, including emulsifiers, antistatic agents, surfactants, viscosity regulators, and solvents in products such as foundation, shaving foam, eye shadow, powder, lipstick, shampoos, and moisturizers [3, 5, 7, 9]. Their hydrophobic and film-forming properties offer additional benefits, such as increased wear, durability, and spreadability [5, 8, 10].

Given the numerous functions of PFAS in cosmetics, direct human exposure through skin contact is significant. Potential exposure pathways include ingestion from lipstick, absorption via the tear ducts through mascara, or dermal absorption through foundation and powder [8, 9, 11]. Their use in cosmetic formulations is still largely unregulated in North America, as well as in many European countries. There is a lack of transparency on labels and many cosmetic products contain PFAS that are not listed on ingredient labels [8]. In addition, PFAS may be unintentionally present as impurities from production processes or as degradation products of larger fluorinated compounds [5, 7, 12].

Research on PFAS in cosmetic products remains limited, but recent studies have confirmed their presence in varying concentrations. For example, Whitehead et al. [8] analyzed 231 cosmetic products from the USA and Canada using particle-induced gamma-ray emission spectroscopy for total fluorine and LC–MS and GC–MS for targeted PFAS detection. Their analysis revealed that 6:2 and 8:2 fluorotelomer compounds — including alcohols, methacrylates and phosphate esters — were most frequently detected, with PFAS concentrations ranging from 22 to 10,500 ng/g. Schultes et al. [1] analyzed 31 cosmetic products from the Swedish market by LC–MS, extractable organic fluorine (EOF) and total fluorine (TF) by combustion ion chromatography (CIC) in five product categories. They identified 25 different PFAS, with perfluorohexanoic acid (PFHxA), perfluoroheptanoic acid (PFHpA), and polyfluoroalkyl phosphate esters (PAPs) frequently detected in foundations and powders. In addition, a recent study by Couteau et al. [3] examined 765 cosmetic products and identified 11 PFAS, with polytetrafluoroethylene (PTFE) present in 25.9% of products and perfluorodecalin in 22.2% of products.

These findings confirm the widespread occurrence of PFAS in personal care and cosmetic products, highlighting the importance of developing precise extraction methods for these complex matrices. Cosmetic products contain a variety of compounds that can interfere with the selective extraction of PFAS, leading to low recoveries or false positives due to contamination or interference from other substances. The most employed method for isolating PFAS from such matrices is solvent extraction, typically involving steps such as ultrasonication, centrifugation, supernatant collection, evaporation, and reconstitution [1, 8] before analysis by LC–MS or GC–MS. However, this approach is not effective for preconcentration, it is poorly selective and poses risks of PFAS loss or contamination during the multiple processing steps.

The aim of this study is to develop alternative and more efficient protocols for the extraction and preconcentration of eight anionic PFAS with different chemical compositions — including per- and polyfluorinated compounds and functional groups such as carboxylates, sulfonates, and phosphate esters — from six different mascara products (both waterproof and non-waterproof). The selected extraction techniques are solid-phase microextraction (SPME) and automated micro solid-phase extraction (µSPE). Optimization of these methods is critical to maximize extraction efficiency without compromising sensitivity, enabling high-throughput analytical solutions for routine analysis. Since cosmetics contain a variety of additives and chemicals with different physicochemical properties, the analysis and isolation of PFAS is a challenge. Therefore, different parameters were optimized for both techniques to ensure the best extraction performance for all analytes. For µSPE, elution conditions—such as the elution solution composition, speed, and volume—were optimized to ensure quantitative recovery of PFAS from the extraction phase. Previously optimized conditions for SPME, including desorption solution, volume, and time [13, 14], were applied, but optimization of the extraction time in mascara was performed to account for the mixed-phase nature of the sample. The dispersive media for mascara was systematically evaluated to optimize the recovery of PFAS compounds. Different compositions of CH_3_OH and H_2_O mixtures were tested for both SPME and µSPE. The mascara sample that showed the least matrix effect was used as a medium for matrix-matched calibration curves and analysis of commercially available mascara samples was performed to determine the presence and concentration of PFAS.

## Experimental section

A detailed description of the standards and other materials used in this work can be found in the Supporting Information (SI) (section S1 Materials). The preparation of the SPME devices followed a protocol from our previous study [13]. The conditions for liquid chromatography and mass spectrometry are described in the SI, section S3.

### SPME procedure

**Fig. 1:**
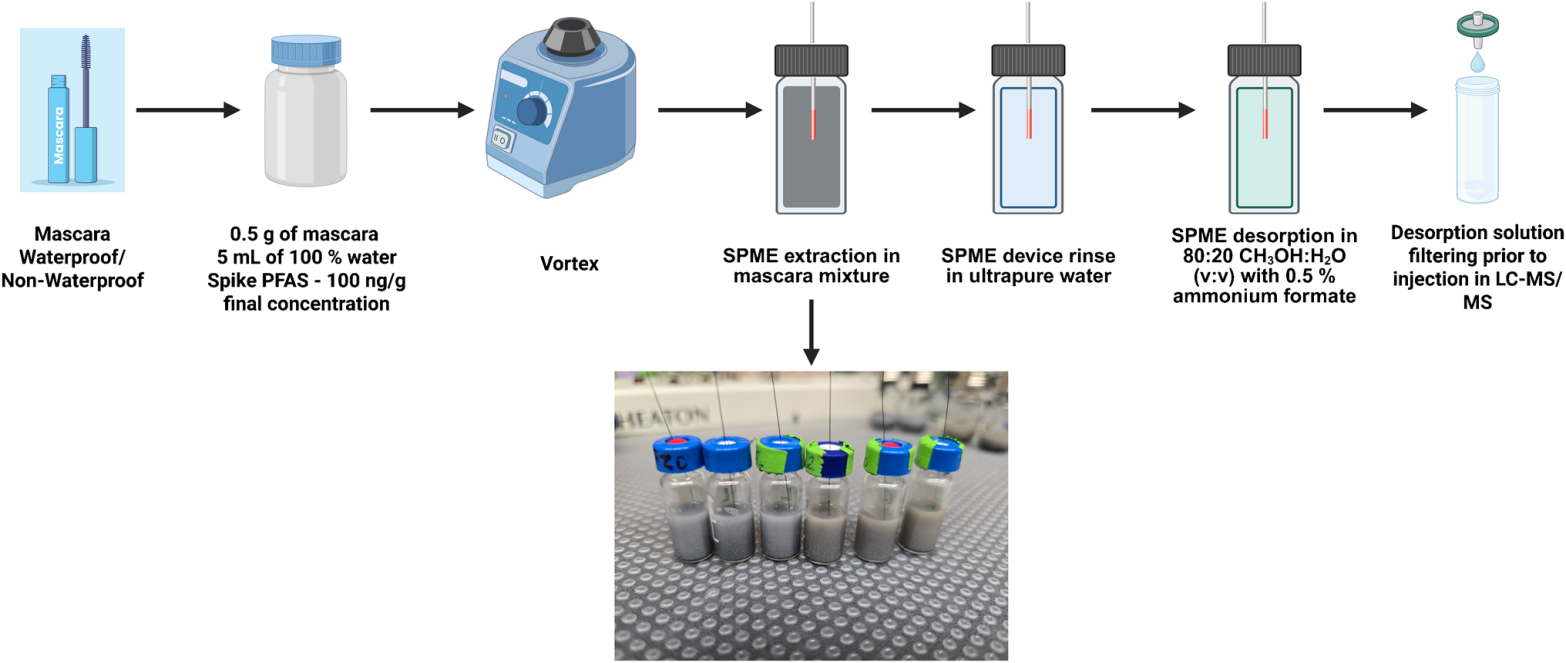
SPME procedure for extraction of PFAS from mascara samples

SPME experimental procedure is shown in Fig. 1. SPME fibers were used for the method optimization and thin film microextraction (TF-SPME) for method validation for improved extraction recoveries. 0.5 g of mascara (waterproof and non-waterproof) was weighed into 10 mL glass vials and spiked 50 µL of a PFAS mixture in CH_3_OH at 1000 ng/mL to achieve a final individual PFAS concentration of 100 ng/g. 5 mL of ultrapure water was added to the spiked mascara and the mixture was vortexed thoroughly. 1.5 mL of the mixture was transferred to a 2 mL glass vial for extraction, which was carried out for 60 min. Desorption was performed in a 500 μL plastic vial with inserts for 20 min with 250 μL of a 80:20 CH_3_OH:H_2_O (v:v) solution containing 2% ammonium formate. After extraction, the SPME devices were quickly rinsed with ultrapure water to remove mascara residues loosely attached to the extraction phase surface, including residual pigments. Both extraction and desorption were carried out at an agitation speed of 1000 rpm. Prior to injection in the LC–MS/MS, the desorption solutions were filtered using a 0.22 µm nylon syringe filters with a 4 mm diameter. The TF-SPME devices were stored in a solution of 50:50 (v/v) CH_3_OH:H_2_O when not in use and rinsed briefly with ultrapure water before extraction.

### Automated µSPE procedure

**Fig. 2:**
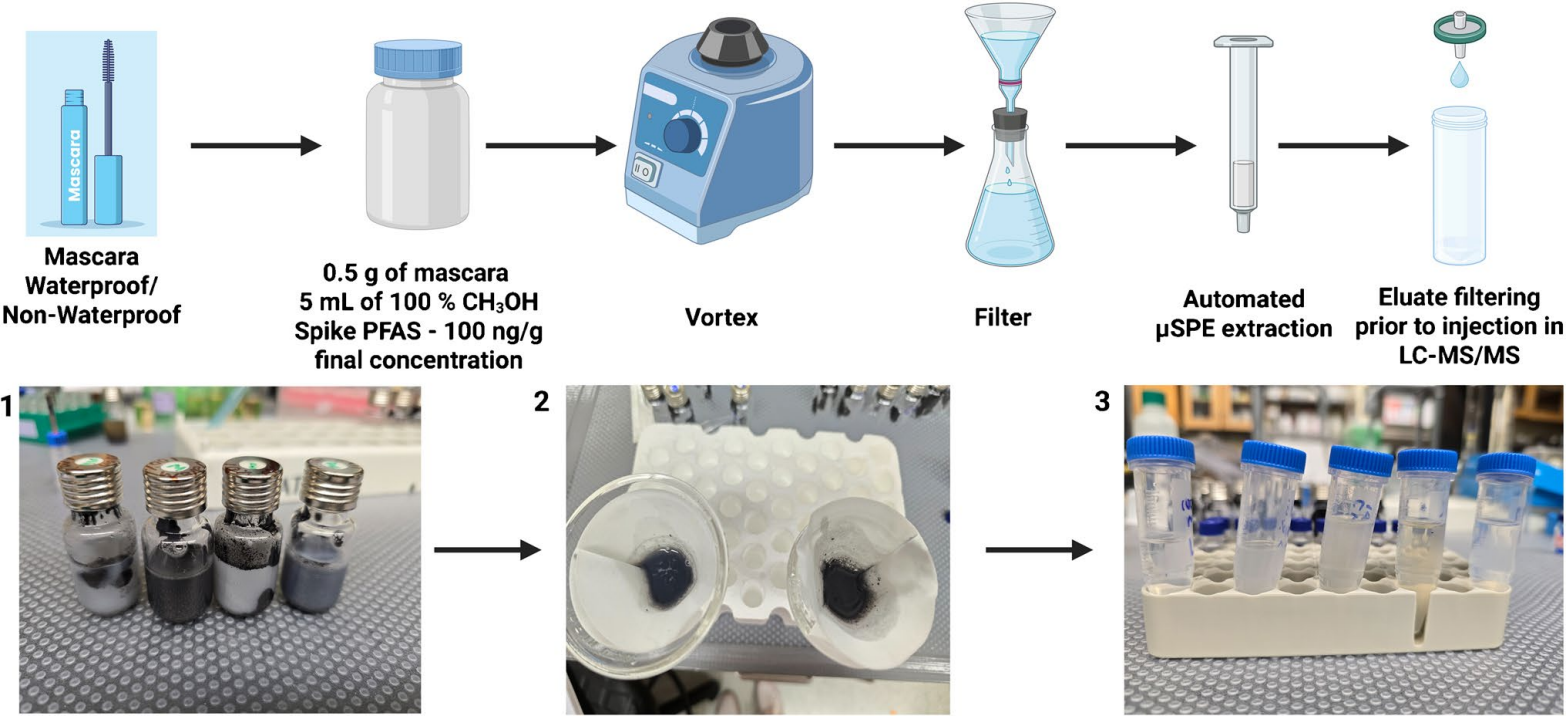
Top: µSPE workflow for extraction of PFAS from mascara sample. Bottom: (1) mascara dispersed in 100% CH_3_OH, (2) filtration of the dispersed sample after vortexing, (3) filtrate collected

0.5 g of mascara was weighed into 10 mL glass vials and spiked with 50 µL of PFAS mixture in CH_3_OH to achieve a final individual concentration of 20 ng/g. 5 mL of CH_3_OH was added to the spiked mascara and the mixture was thoroughly vortexed to ensure homogeneity. The solution was then filtered using a Whatman filter paper (grade 1) with a diameter of 110 mm and the filtrate was collected for preconcentration. The µSPE procedure was automated using a PAL System and PAL System µSPE WAX cartridges (CTC Analytics AG, Zwingen, Switzerland). Before extraction, the µSPE WAX cartridges were conditioned with 400 µL ultrapure water at a dispensing rate of 5.0 µL/s. Subsequently, 400 µL of the filtered sample was loaded on the cartridge at the same dispensing rate. Elution was performed with 100% methanol containing 0.5% ammonium formate at 1 µL/s. Finally, 100 µL of water was added to the eluate to achieve a final solvent composition of 80:20 CH_3_OH:H_2_O (v:v) and filtered using a 0.22 µm nylon syringe filters with a 13 mm diameter. Figure 2 illustrates the experimental procedure. Details on the automated PAL System procedure are discussed in the SI Section S4, and a schematic image of the PAL System is shown in Fig. S1.

### Matrix effect and method validation

To assess the matrix effect for both SPME and µSPE, extractions were first performed on different mascara samples. After desorption/elution, a PFAS mixture was spiked into the solution at a concentration of 10 ng/mL before instrumental analysis. The PFAS mixture was also added directly to the neat desorption/elution solution and analyzed. The matrix effect was evaluated by comparing the peak area ratio between the post-desorption spiked mascara samples and the peak area from the neat desorption/elution solution using Eq. 1. Values between 70 and 130% were considered acceptable. Values exceeding 130% indicated signal enhancement, while those below 70% indicated signal suppression. The discussion on the matrix effect for µSPE and SPME is found in Sections 5 and 6 of the SI.

Matrix-matched calibration curves were performed for both the SPME and µSPE methods using P4 W mascara, which showed the minimal matrix effect. Final individual concentration levels of PFAS spiked in the mascara used for the calibration curve included 0.025, 0.05, 0.1, 0.2, 0.5, 1, 1.5, 5, 10, 20, and 25 ng/g and the internal standards concentrations were 0.25 ng/g. Accuracy and precision over a period of 1 week were evaluated at 0.25, 0.75, 2, and 7.5 ng/g. The limits of detection (LOD) and limits of quantification (LOQs) were evaluated, respectively, as the lowest concentration of the analyte generating a chromatographic peak with S/N ≥ 3, and the lowest point of the calibration providing accuracy between 70 and 130% with relative standard deviation (RSD) ≤ 20%.

1 $$\text{ME}{\%}\;=\;\frac{Peak\;area\;of\;the\;post\;extraction\;spiked\;desorption\; (elution)\;solution}{Peak\;area\;of\;the\;desorpton\;( elution)\;solution}\;\times\;100$$

## Results and discussion

### µSPE optimization

#### Extraction phase chemistry

**Fig. 3:**
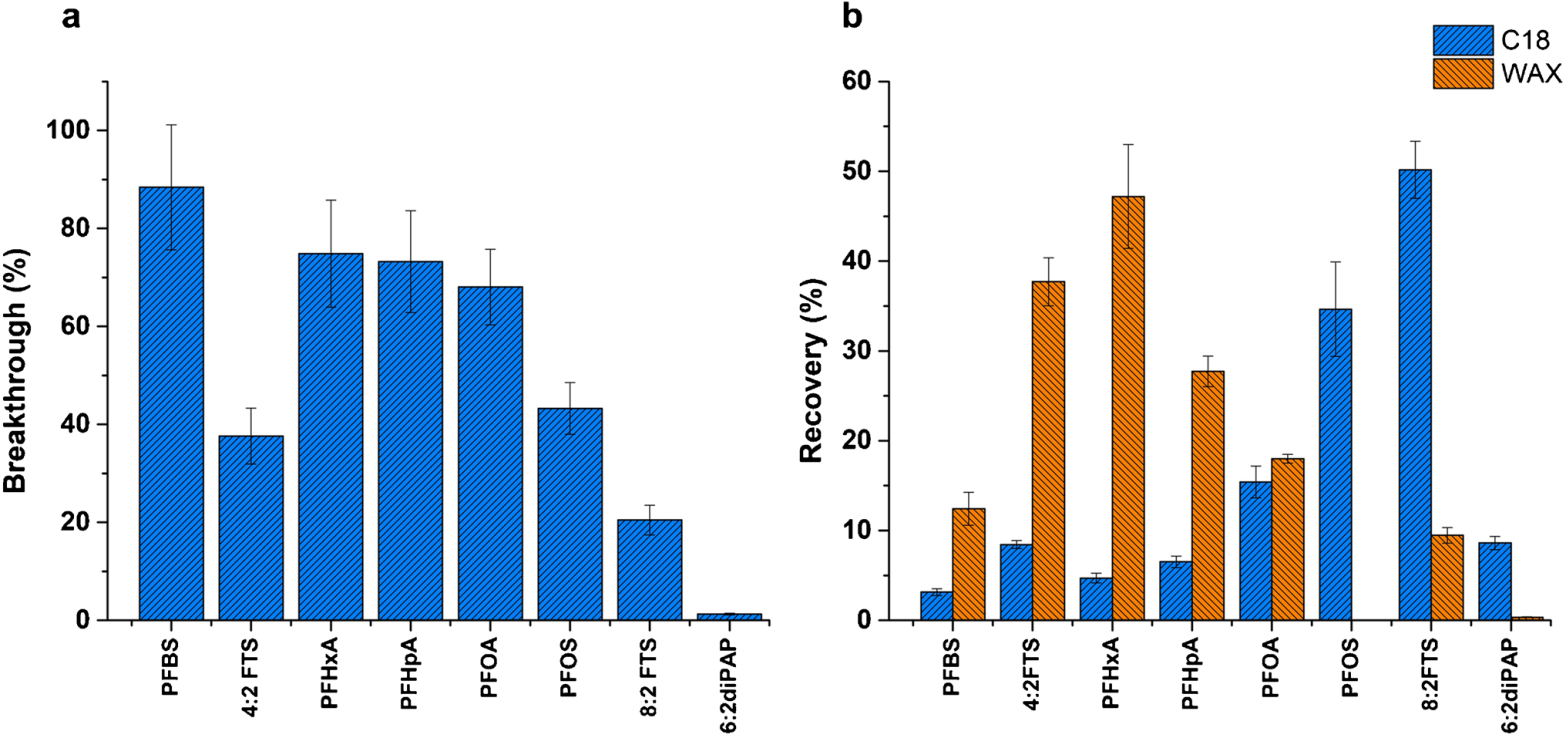
Comparison of C18 and WAX sorbents for the extraction of PFAS via µSPE. **a** Percent breakthrough of PFAS from the C18 cartridge and **b** percent recovery of the PFAS retained on both cartridges. Loading solution: 10 ng mL^−1^ PFAS in 400 µL ultrapure water. Elution solution: 400 µL 80:20 CH_3_OH:water (v:v) with 0.5% ammonium formate

The chemistry of the sorbent used in any solid-phase extraction process is critical for optimizing extraction efficiency and recovery of analytes from complex mixtures. Eight anionic PFAS were selected in this work with a diverse range of chemistries, including both short- and long-chain perfluorinated compounds (PFBS, PFHxA, PFHpA, and PFOS) as well as short- and long-chain polyfluorinated compounds (4:2 FTS and 8:2 FTS). 6:2 diPAP was also selected based on its frequent detection in cosmetics and potential for biotransformation into perfluoroalkyl carboxylic acids (PFCAs) [15–17]. These PFAS have various functional groups, such as carboxylates, sulfonates and phosphates. In this study, two extraction phase chemistries, octadecyl silica (C18) and weak anion exchange (WAX), were investigated, and their extraction performance was assessed. During the µSPE protocol, the sample effluent collected immediately after loading onto the cartridge was analyzed to assess potential PFAS breakthrough during the loading step. The results showed breakthrough of PFAS analytes with the C18 sorbent, while no analytes were detected in the loading fraction obtained from the WAX sorbent. The breakthrough of PFAS through the C18 sorbent was observed to decrease with increasing chain length, indicating that longer-chain PFAS have stronger interactions with the C18 sorbent compared to shorter-chain compounds (Fig. 3a). The analysis of the elution solution from the C18 sorbent revealed the highest recoveries for PFOS (36%) and 8:2 FTS (49%) (Fig. 3b), while showing poor extraction efficiency for shorter-chain PFAS, such as PFBS, 4:2 FTS, PFHxA, and PFHpA. These findings confirm strong hydrophobic interactions between the long-chain PFAS and the C18 sorbent, with increasing carbon chain length corresponding to greater hydrophobicity [13, 14].

For the WAX sorbent, all analytes except PFOS and 6:2 diPAP were recovered (Fig. 3b). The higher recovery of short-chain PFAS compared to the more hydrophobic compounds on the WAX cartridge is due to stronger electrostatic interactions with the positively charged WAX moiety. The strength of the electrostatic interaction between the WAX moiety and the PFAS depends on the relative charge distances, which leads to stronger interactions with shorter-chain PFAS [13]. This observation is consistent with previous studies demonstrating the effectiveness of WAX sorbents for shorter-chain PFAS compared to C18 [13, 14, 18]. Due to the low recovery of the shorter-chain PFAS and the significant breakthrough observed with C18, WAX sorbents were selected for further optimization in this study. The optimization of the elution conditions is necessary to improve the recovery of hydrophobic PFAS such as PFOS and 6:2 diPAP as discussed in "Elution conditions" section.

#### Elution conditions

**Fig. 4:**
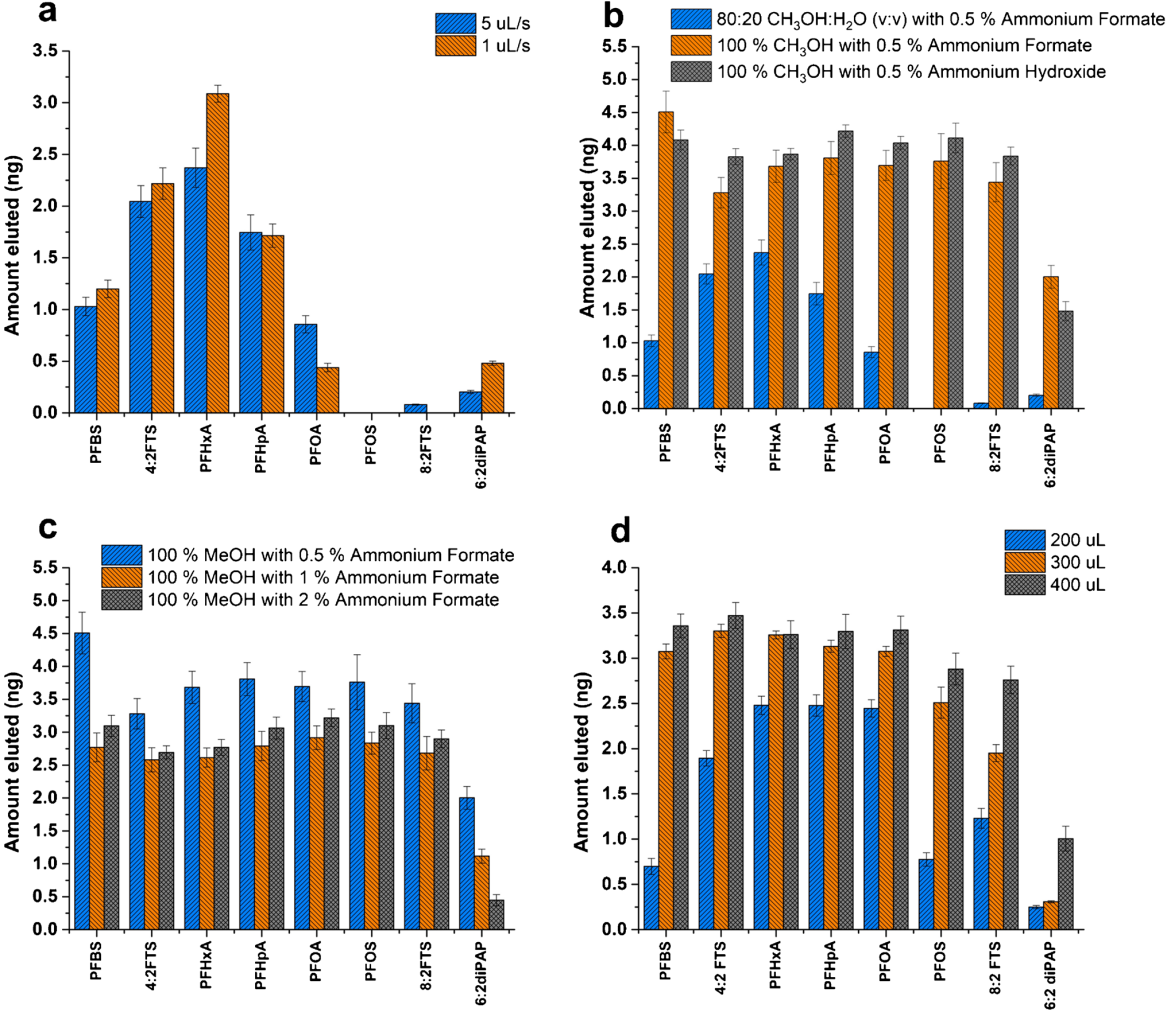
Optimization of the elution conditions with WAX cartridge. **a** Elution speed, **b** desorption solution and additive, **c** ammonium formate percentages, and **d** elution volume

To enhance the recovery of the targeted PFAS, particularly PFOS and 6:2 diPAP, the elution conditions were optimized. First, elution speeds of 1 µL/s and 5 µL/s were evaluated using the autosampler’s automated solvent delivery system to ensure adequate sample residence time within the cartridge, promoting effective interaction between the extraction phase and PFAS. For all PFAS except PFHxA, PFOA, and 6:2 diPAP, there was no significant difference in the amount eluted when the elution speed was changed from 5 µL/s to 1 µL/s (Fig. 4a). Further optimization was performed by evaluating the composition of the elution solution. The addition of an ammonium salt to the elution solution is critical for a quantitative elution of PFAS when using WAX extraction phases containing primary/secondary amine functional groups. Our previous studies involving hydrophilic-lipophilic balance weak anion exchange (HLB-WAX) and fluorinated WAX sorbents have shown that the desorption mechanism is highly dependent on the presence of additives in solution that facilitate anion exchange with sorbed PFAS [13, 14, 19]. Therefore, varied concentrations of ammonium formate and ammonium hydroxide were evaluated to improve PFAS elution from the WAX sorbent. Initially, the elution solution used was 80:20 CH_3_OH:H_2_O (v:v) with 0.5% ammonium formate (Fig. 4b). However, to achieve quantitative elution in a single step, stronger elution solutions were tested, specifically 100% MeOH with 0.5% (w/v) ammonium formate and 100% MeOH with 0.5% (v/v) ammonium hydroxide. Results showed that 100% MeOH containing either ammonium formate or ammonium hydroxide significantly enhanced the recovery of all PFAS compounds compared to the 80:20 (v/v) MeOH:H_2_O solution, yielding up to a twofold increase in recovery. Moreover, it was also observed that when elution solutions containing ammonium hydroxide were analyzed, the chromatographic peaks appeared distorted, especially for the early eluting analytes (Fig. S2). Therefore, ammonium formate remained the optimal choice as an additive for elution. The effect of ammonium formate concentration in the elution solution on the recovery was also investigated at 0.5%, 1%, and 2% (v:v). The results indicated a decrease in the amount eluted as the concentration of ammonium formate increased (Fig. 4c). Therefore, 0.5% ammonium formate was selected as the best concentration of additive in the elution solution.

Elution volumes of 200 µL, 300 µL, and 400 µL were evaluated to optimize the volume required for efficient desorption and preconcentration (Fig. 4d). For all analytes, recovery improved with an increasing elution volume. While some analytes reached a plateau at 300 µL, the recovery of others continued to increase up to 400 µL. For example, the recovery of PFBS increased from 17 to 84% as the volume increased from 200 µL to 400 µL. Larger elution volumes were essential for optimal PFAS recovery, as carryover after the second elution was minimal. At 200 µL, the carryover was > 20% for all analytes, but at 400 µL it dropped to < 2%. Therefore, 400 µL was chosen as the optimal elution volume for maximum recovery. Volumes greater than 400 µL were not tested to ensure sufficient preconcentration.

### Effect of sample matrix composition and sonication on recovery

Optimizing the composition of the sample matrix is crucial to maximizing analyte recovery. Control solutions (dispersive media without mascara) with varying CH_3_OH:H_2_O compositions were evaluated to assess PFAS recovery. The compositions tested included 100% ultrapure H_2_O, 90:10 (v/v) H_2_O:CH_3_OH, 50:50 (v/v) H_2_O:CH_3_OH, 20:80 (v/v) H_2_O:CH_3_OH, and 100% CH_3_OH. As shown in Fig. S3a, the amount of PFAS retained increased at higher CH_3_OH content, especially for 6:2 diPAP. Additional tests were conducted to assess the impact of sonication on PFAS recovery. Sonication of complex samples prior to sample loading, such as mascara, can be useful to improve recovery for the targeted analytes. To this end, solutions of 100% H_2_O, 50:50 (v/v) H_2_O:CH_3_OH, and 100% CH_3_OH were used to evaluate the effect across different solvent compositions. As shown in Fig. S3b, sonication resulted in lower recoveries for several PFAS compounds at higher water content, with 6:2 diPAP being particularly affected. The marked decrease in 6:2 diPAP recovery at elevated H_2_O levels may be attributed to hydrolysis of the phosphate ether bonds in aqueous media, leading to the formation of 6:2 FTOH and 6:2 monoPAP as intermediates, which can subsequently degrade into carboxylic acids [15–17].

**Fig. 5:**
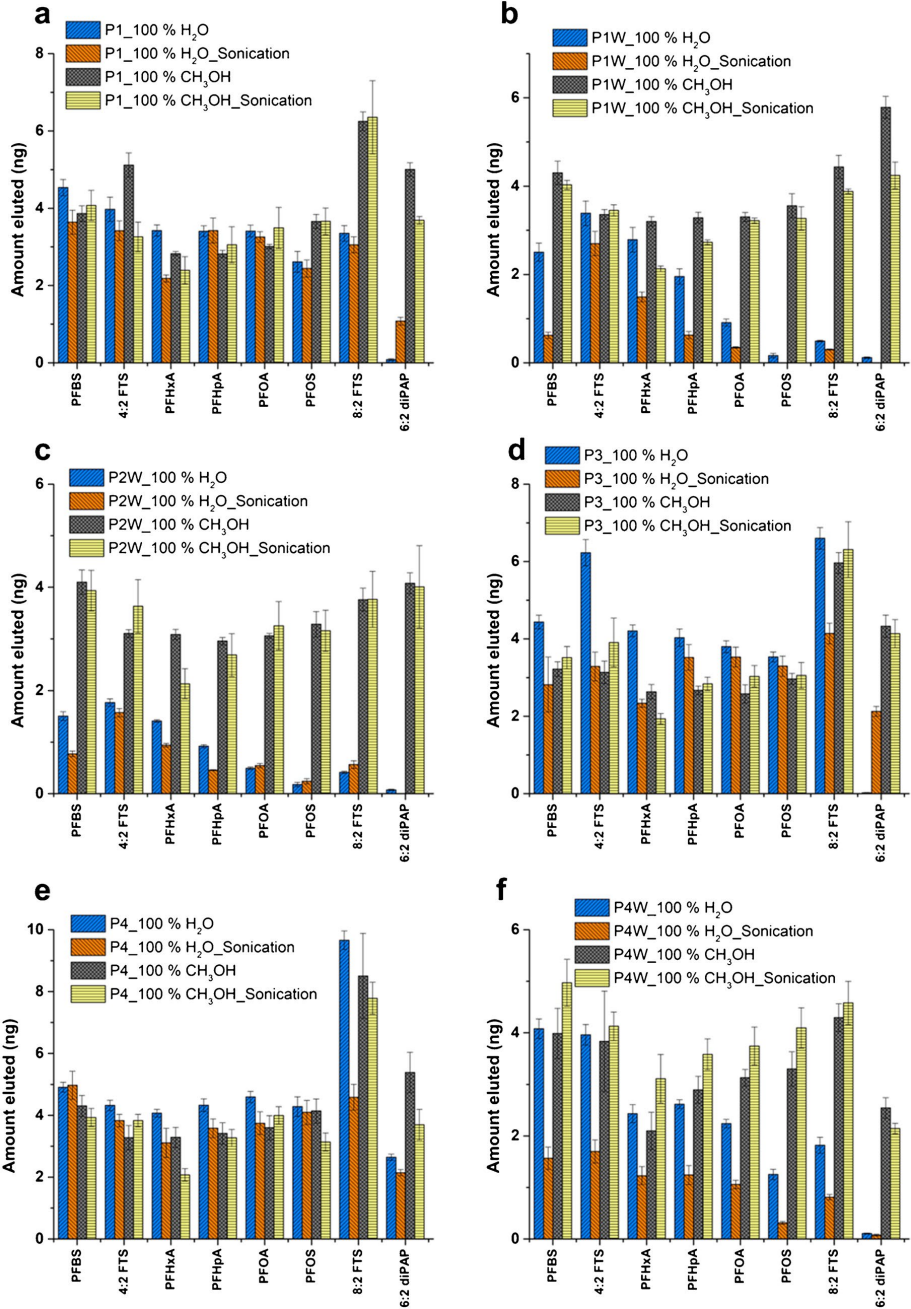
µSPE dispersive media optimization with sonication and no sonication of PFAS in six different mascara types: **a** P1, **b** P1 W, **c** P2W, **d** P3, **e** P4, and **f** P4W

Given that PFAS recovery was highest at elevated organic content, 100% CH_3_OH was selected as the dispersive medium for the mascara samples. To verify the consistency of these findings across various mascara formulations and brands, six different mascara products were evaluated: product 1 (P1), product 1 waterproof (P1 W), product 2 waterproof (P2 W), product 3 (P3), product 4 (P4), and product 4 waterproof (P4 W). 100% H_2_O and 100% CH_3_OH were reassessed as a dispersive medium to test the behavior of PFAS, particularly in waterproof mascaras. Sonication of the dispersive medium was also evaluated to determine whether the presence of the mascaras stabilizes PFAS in 100% H_2_O. As shown in Fig. 5, sonication had a limited impact on PFAS recovery from non-waterproof mascaras but significantly affected recovery from waterproof formulations. A pronounced decrease in 6:2 diPAP recovery following sonication with 100% H_2_O was consistently observed, regardless of mascara type. Overall, PFAS recovery from waterproof mascaras using 100% H_2_O was low across all analytes, particularly in samples P1 W and P2 W. In contrast, optimal recovery for all PFAS—including 6:2 diPAP—was achieved using 100% CH_3_OH, with or without sonication. These results confirm that 100% CH_3_OH is the most effective dispersive medium for extracting PFAS from complex mascara matrices.

### µSPE: matrix effect and recovery

Matrix effects for the six mascara products were assessed, with values between 70 and 130% considered acceptable based on the U.S. EPA accuracy criteria for PFAS analysis [2–4]. Significant signal enhancement was observed for 6:2 diPAP for all mascaras except P4 W. For 8:2 FTS, signal enhancement (282%) occurred in P4, while signal suppression for PFHpA (67.4%) was observed in P1 (Table S4). All other analytes were within the acceptable range. To reduce the potential of matrix effect occurrence for 6:2 diPAP, a washing step with water was performed after loading the cartridges with the samples. This step was intended to remove matrix components prior to PFAS elution. Various water volumes (200, 300, and 400 µL) were tested, as shown in Fig. S5, and no significant differences in PFAS recovery were observed across the tested volumes. Fig. S5 also compares recoveries with and without the wash step prior to elution, revealing that the inclusion of a washing step results in a significant loss of PFAS. Matrix effect was reevaluated by incorporating the washing step in the analytical workflow and using 400 µL ultrapure water as the wash volume. The results revealed the inefficiency of the wash step in mitigating matrix effects, especially for 6:2 diPAP, unless signal correction with isotopically labelled internal standards is performed (Table S5). To further validate the method and obtain a matrix-matched calibration curve, P4W was selected as it showed the least matrix effects. PFAS absolute recovery for the µSPE protocol under optimized conditions was calculated using Eq. 2, proposed by Matuszewski et al. [5] (Table 1).

**Table 1:** Absolute PFAS recovery using the optimized µSPE protocol across six different mascara products

	Absolute recovery (%)					
	Product 1	Product 1 Waterproof	Product 2	Product 3	Product 4	Product 4 Waterproof
PFBS	112.5 ± 12.0	100.6 ± 14.6	119.4 ± 6.2	149.8 ± 11.1	74.9 ± 11.1	116.7 ± 14.9
4:2 FTS	107.0 ± 11.7	92.4 ± 15.6	116.7 ± 3.5	82.4 ± 16.1	71.9 ± 14.4	128.9 ± 11.4
PFHxA	104.6 ± 14.6	93.2 ± 14.6	112.5 ± 3.6	71.9 ± 14.8	61.0 ± 15.2	125.9 ± 13.0
PFHpA	119.5 ± 15.4	98.0 ± 14.2	116.0 ± 4.0	77.4 ± 17.2	68.0 ± 17.6	108.7 ± 15.6
PFOA	114.2 ± 12.6	93.1 ± 15.6	87.6 ± 6.1	72.0 ± 12.9	63.3 ± 14.4	104.0 ± 15.7
PFOS	112.4 ± 12.1	85.5 ± 15.1	116.0 ± 8.3	87.8 ± 9.5	74.7 ± 15.0	97.7 ± 13.4
8:2 FTS	140.8 ± 13.4	92.0 ± 19.0	122.0 ± 5.5	89.8 ± 9.1	85.3 ± 14.3	112.6 ± 16.3
6:2 diPAP	120.9 ± 15.1	119.6 ± 12.1	234.1 ± 2.7	85.8 ± 16.7	87.9 ± 17.9	152.3 ± 15.7

Number of replicates *n* = 6 (± standard error)

2 $$\text{Recovery}\;=\;\frac{Peak\;area\;of\;standard\;spiked\;before\;extraction}{Peak\;area\;of\;standard\;spiked\;after\;extraction}\;\times\;100\;{\%}$$

### SPME optimization

**Fig. 6:**
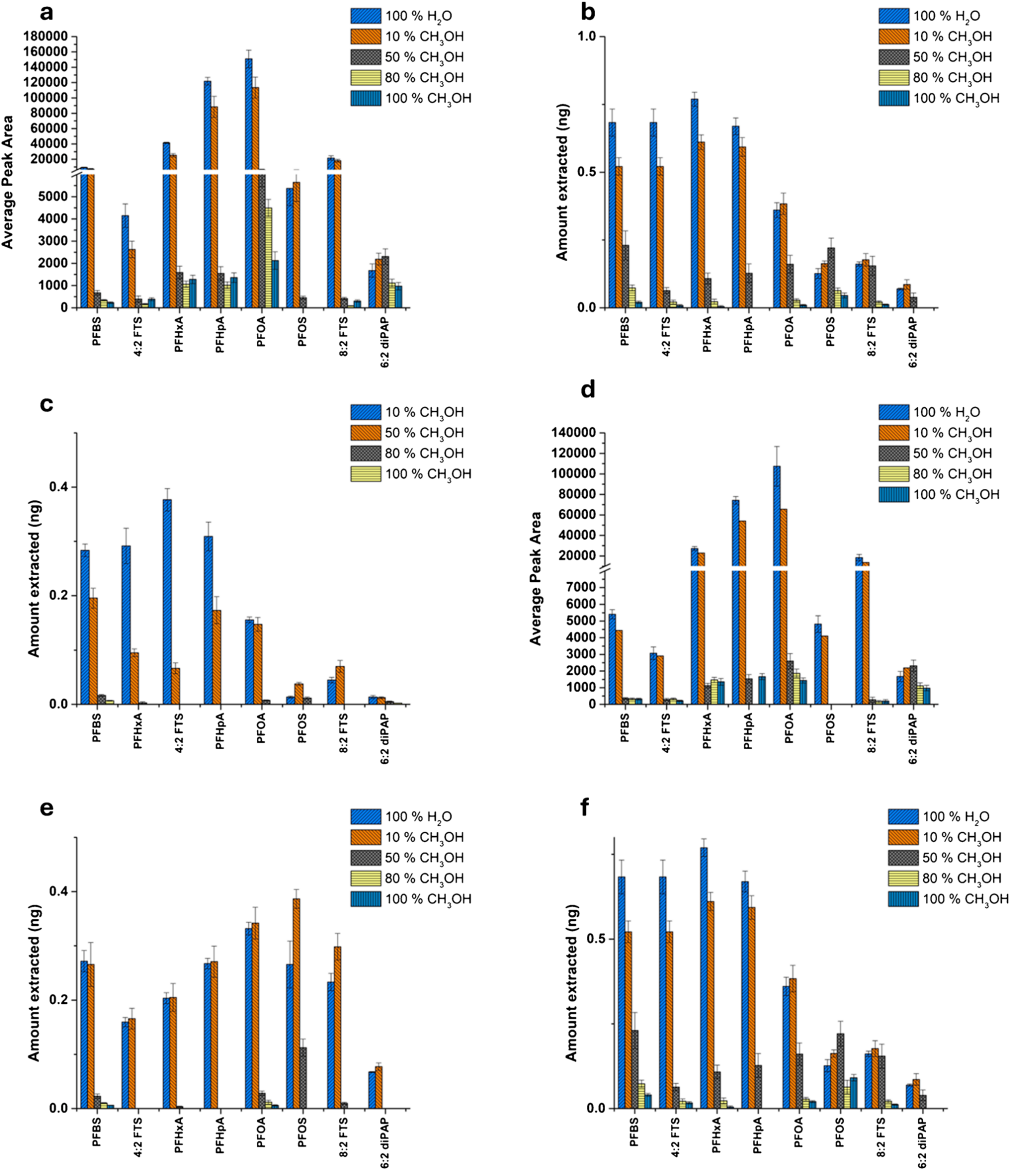
Optimization of dispersive media for SPME using varying H_2_O:CH_3_OH ratios—100% H_2_O, 90:10, 50:50, 20:80, and 100% CH_3_OH (v/v). Results are shown for **a** P1, **b** P1W, **c** P2W, **d** P3, **e** P4, and **f** P4W

For the SPME method, only minimal optimization was necessary, as previously established parameters from our earlier PFAS studies were applied in this work [13, 14]. The applied parameters included the extraction phase chemistry (HLB-WAX/PAN), a desorption solution composed of 80:20 (v/v) CH_3_OH:H_2_O containing 2% ammonium formate, a desorption volume of 250 µL, and a desorption time of 20 min. The optimal dispersive media composition for the SPME technique was assessed similarly to the µSPE method. For all mascara types, the amount of PFAS extracted decreased with increasing CH_3_OH content (Fig. 6). This trend was also noticed in our previous work [19] where the amount of PFAS extracted decreased as the organic content (methanol or acetonitrile) increased indicating the suppression of hydrophobic interaction between the PFAS and fluorinated WAX sorbent [19]. As shown in Fig. 6, all mascara products exhibited low recoveries for long-chain PFAS, including PFOS, 8:2 FTS, and 6:2 diPAP. This trend suggests that these more hydrophobic compounds may interact strongly with mascara components, limiting their availability for extraction via SPME. Based on these results, 100% H_2_O was selected as the optimal dispersive medium for PFAS extraction. Additionally, consistent with µSPE findings, 6:2 diPAP recovery was negatively impacted by sonication in 100% H_2_O. To address this, sonication was excluded from the final optimized protocol to improve recovery of 6:2 diPAP. The extraction time profile was evaluated in mascara dispersed in 90% H_2_O (P4W), as PFAS show a different kinetic behavior in a complex matrix than in simpler matrices such as pure water. The extraction time profile was performed from 10 to 120 min using TF-SPME for enhanced extraction efficiency (Fig. S4) with equilibrium of the analytes achieved at 90 min. No significant differences were observed in the amounts of PFOS and 6:2 diPAP extracted with increasing extraction time, possibly indicating interactions between these compounds and mascara components. This may also be attributed to poor extraction affinity with the sorbent. For this study, 60 min was selected as the optimal extraction time to find a compromise between extraction efficiency and sample throughput.

### SPME matrix effect

SPME is known for minimizing or eliminating matrix effects in complex samples due to the biocompatibility of the extraction phase, which reduces the co-extraction of interfering components [6, 7]. Direct immersion of the SPME fiber and thin film into the complex mascara matrix was enabled by the use of polyacrylonitrile (PAN) as a biocompatible binder. This biocompatibility feature is absent in µSPE cartridges. Additionally, SPME offers the advantage of integrating sample preparation, preconcentration, and isolation into a single step, minimizing the risk of cross-contamination associated with the multiple handling steps required in µSPE. Minimal matrix effects (Table 2) were observed using SPME in this study, with signal enhancement detected for 4:2 FTS in P1W, 8:2 FTS in P4, and 6:2 diPAP in P2W. Matrix effects for other analytes remained within the acceptable range of 70–130%.

**Table 2:** Matrix effect calculated for the SPME protocol

SPME matrix effect (%)						
Analytes	Product 1	Product 1 Waterproof	Product 2	Product 3	Product 4	Product 4 Waterproof
PFBS	86.4 ± 12.6	92.9 ± 13.5	114.8 ± 10.5	119.1 ± 12.5	108.5 ± 11.1	111.9 ± 9.5
4:2 FTS	123.5 ± 23.0	151.9 ± 13.1	112.9 ± 9.6	116.8 ± 11.7	110.0 ± 11.8	107.0 ± 8.6
PFHxA	83.5 ± 13.3	92.9 ± 13.2	114.2 ± 12.3	120.0 ± 13.2	113.5 ± 13.3	111.0 ± 10.6
PFHpA	123.4 ± 12.1	83.4 ± 10.8	113.2 ± 10.6	119.0 ± 12.1	110.8 ± 11.5	107.9 ± 8.6
PFOA	124.2 ± 9.9	102.5 ± 14.6	112.1 ± 9.4	116.7 ± 11.8	110.1 ± 12.1	108.6 ± 8.9
PFOS	116.6 ± 13.3	85.8 ± 11.0	111.4 ± 15.0	112.5 ± 15.4	106.8 ± 14.3	103.3 ± 12.2
8:2 FTS	84.2 ± 15.4	90.8 ± 13.4	119.8 ± 14.9	128.4 ± 14.6	131.7 ± 18.0	130.6 ± 13.2
6:2 diPAP	90.3 ± 16.7	98.0 ± 18.1	135.2 ± 16.3	121.6 ± 17.0	125.6 ± 17.4	108.6 ± 12.2

Number of replicates *n* = 9 (± standard error)

### Method validation — µSPE and SPME

The overall performance of the two extraction techniques was evaluated by performing matrix-matched calibration curves for all analytes using P4 W, which showed minimal matrix effects for all compounds. A concentration range between 0.025 and 25 ng/g was investigated for linearity and internal standards were spiked at 0.25 ng/g. For the SPME method, matrix-matched calibration curves were performed using 100% H_2_O dispersive media for the mascara, whereas for the µSPE method 100% CH_3_OH was used as dispersive media. A wide linear dynamic range for all analytes was achieved with both methods, as summarized in Table 3.

**Table 3:** Figures of merit for SPME and µSPE methods

Analyte	SPME linear dynamic range (ng/g)	Weighting	R^**2**^	µSPE linear dynamic range ng/g	R^**2**^	Weighting
PFBS	0.025–25	1/x	0.995	0.1–25	0.990	1/x
4:2 FTS	0.05–25	1/x	0.996	0.05–25	0.996	1/x
PFHxA	0.05–25	1/x	0.998	0.025–25	0.996	1/x
PFHpA	0.1–25	1/x	0.983	0.05–25	0.995	1/x
PFOA	0.05–25	1/x	0.999	0.025–25	0.994	1/x
PFOS	0.5–25	1/x	0.986	0.2–25	0.988	1/x
8:2 FTS	0.5–25	1/x	0.965	0.1–10	0.992	1/x
6:2 diPAP	1–25	1/x	0.975	0.5–25	0.986	1/x

The SPME method achieved lower limits of quantification (LOQ) for the most hydrophilic PFAS tested, namely PFBS (LOQ = 0.025 ng/g). However, as the analytes became more hydrophobic (PFHpA to 6:2 diPAP), their LOQs also increased, with the exception of PFOA, which had an LOQ of 0.05 ng/g. More hydrophobic compounds, namely PFOS, 8:2 FTS, and 6:2 diPAP, achieved LOQs within 0.5 to 1 ng/g. In contrast, the µSPE method produced a higher LOQ for PFBS (0.1 ng/g) but outperformed SPME for hydrophobic PFAS, achieving LOQs of 0.2 ng/g for PFOS, 0.1 ng/g for 8:2 FTS, and 0.5 ng/g for 6:2 diPAP. The LOQ for 4:2 FTS was consistent across both methods. When using µSPE, signal saturation for 8:2 FTS occurred at concentrations above 10 ng/g. In general, these results show that both methods are suitable for the extraction of PFAS from mascara, although µSPE performs better for the hydrophobic PFAS as extraction can be performed with 100% MeOH. Accuracy and reproducibility were studied for µSPE (Table S6) and SPME (Table S7) for a period of 7 days. All analytes showed good accuracy and precision with acceptable values between 70 and 130%, except 8:2 FTS and 6:2 diPAP, which showed high accuracy values after day 1.

### Analysis of real samples

**Fig. 7:**
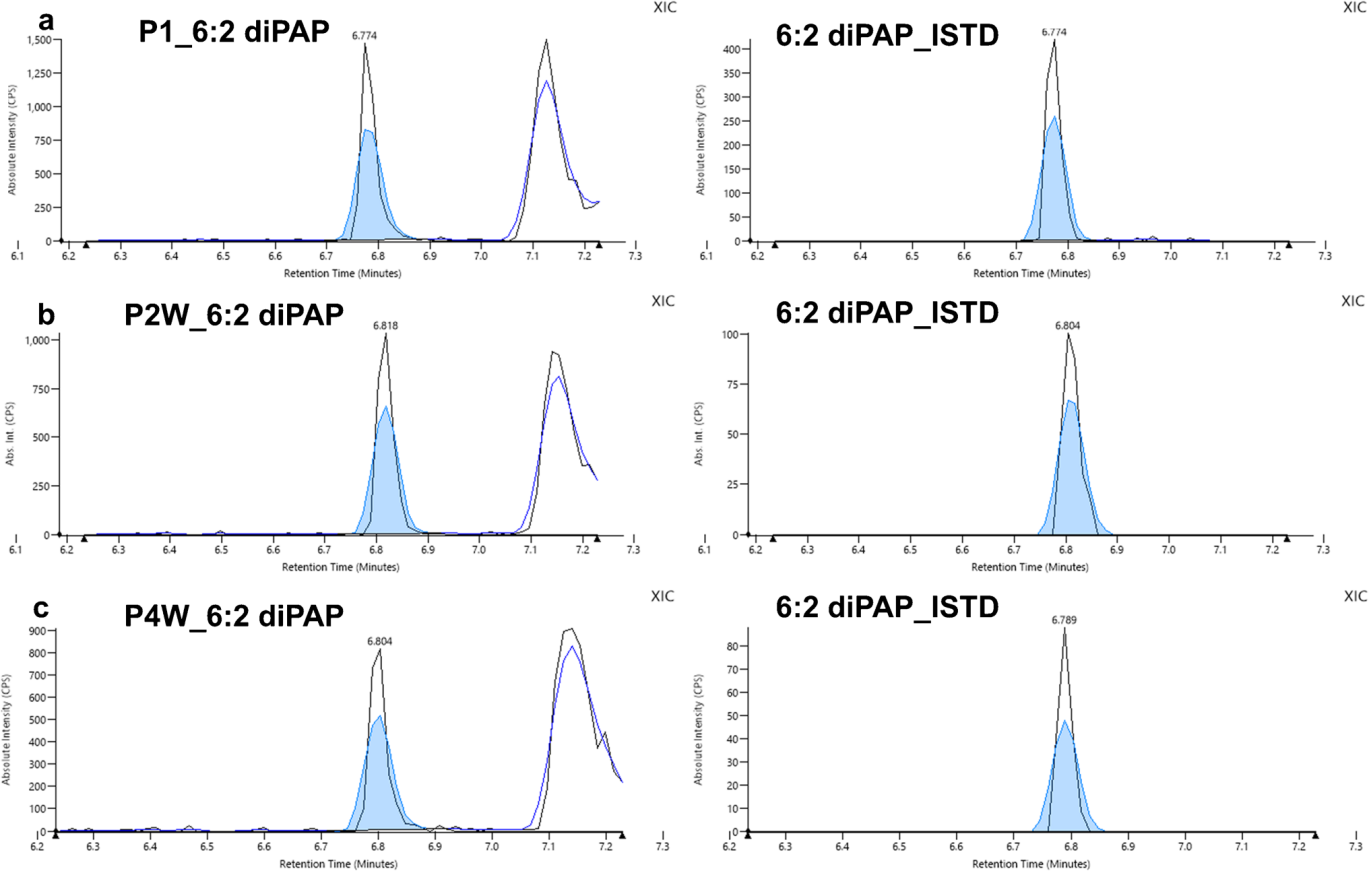
Selected chromatograms of 6:2 diPAP and its internal standard from the analysis of the real sample in mascara: **a** P1, **b** P2W, and **c** P4W

Extractions using SPME and µSPE methods were performed on nine mascara samples manufactured in different countries, including the USA, Italy, Luxembourg, and Russia. PFOA was detected in six of the nine samples using SPME, with the highest concentration found in P2 W at 3.04 ± 0.33 ng/g, while the others were below the LOQ. PFOA was < LOQ in P7 using the µSPE method. 6:2 diPAP was detected in four of the nine mascara samples tested for both methods. For the SPME method, the concentrations of P1 and P7 were below the < LOQ, while the concentrations of P2W and P4W were 2.21 ± 0.19 ng/g and 1.38 ± 0.19 ng/g, respectively. Using the µSPE method, P2W showed the highest concentration at 3.48 ± 0.44 ng/g, followed by P4 W and P1 with concentrations of 1.36 ± 0.17 ng/g and 1.26 ± 0.19 ng/g, respectively. In P7, 6:2 diPAP was detected, but its concentration was below the limit of quantification (LOQ). Selected chromatograms of 6:2 diPAP detected in the mascaras are presented in Fig. 7. Tables S8 and S9 summarize the presence of PFAS in the mascaras, analyzed using both µSPE and SPME techniques. The variations in concentrations observed between the two methods can be attributed to differences in the matrix composition of the various mascaras, as matrix-matched calibration was performed using P4W, in fact the concentrations obtained for 6:2 diPAP in P4W using the two methods are comparable within error. Based on the results, accurate quantification requires matrix-matched calibration using the same type of mascara being analyzed, provided that blank samples (free of target analytes) are available. Alternatively, a standard addition calibration approach should be employed to further minimize matrix effects. Although the concentrations found in this study are lower than those reported in the literature for other cosmetic products [1, 8, 12, 20], the presence of PFAS in cosmetics is still evident. Given the wide range of personal care and cosmetic products, many categories of cosmetics still need to be investigated. In addition, mascaras from different regions of the world have been observed to contain PFAS, suggesting that regulatory measures are still inadequate.

Furthermore, to ensure that the SPME and µSPE protocols were free of PFAS contamination, blank extractions were performed on the TF-SPME and µSPE cartridges using either 100% H_2_O or 100% CH_3_OH. These blanks demonstrated the absence of all analytes studied except for traces of PFOA and 6:2 diPAP from the SPME and µSPE protocols. Blank subtractions were performed for the quantification of PFAS in the real samples to get accurate concentrations. Instrument and solvent blanks were also constantly monitored to ensure the system was free from PFAS interferences.

### Greenness assessment

Sample preparation is widely regarded as the most important step in any analytical process, as it significantly influences the overall success of the analytical workflow. It is crucial for isolating and enriching the target analytes, removing interfering matrices and ensuring that the final sample composition is compatible with the analytical techniques [21]. Armenta et al. [22] emphasize that sample preparation remains a key challenge to the progress of green analytical chemistry due to factors such as the use of solvents, reagents, acids, or bases for pH adjustment, energy consumption and consumables (e.g., cartridges, pipette tips). Nevertheless, it is often impractical to skip this step altogether, as many samples cannot be analyzed in their original form through direct injection into analytical instrumentation. This section provides an objective comparison of the two methods used in this study — SPME and µSPE — and suggests possible improvements to enhance their applicability and sustainability. Analytical greenness metric for sample preparation (AGREEprep) [21] and blue applicability grade index (BAGI) [23] assessment tools were applied to these methods. AGREEprep overall scores fall between 0 and 1 where values of 0 means the worst performance in all criteria and overall score of 1 signifies best performance. The value obtained for SPME was 0.51 and µSPE was 0.44 (Fig. S6). A BAGI score of 75.0 and 70.0 were assigned to SPME and µSPE respectively (Fig. S7).

The main differences between these methods are sample throughput, reusability, and sustainability. SPME offered a higher throughput, as it was able to process up to 40 to 50 samples simultaneously in one hour using an agitator. In contrast, the automated µSPE is limited to processing four samples per hour, although it requires no user intervention after loading samples and cartridges in the autosampler. Reusability and sustainability are important aspects as they help to conserve resources and minimize waste. SPME devices are highly durable and reusable over long periods of time, whereas µSPE cartridges are single use. Both methods enabled preconcentration without further evaporation to dryness and sample reconstitution needed for the desorption/elution solution. A key advantage of SPME is the ability to sample directly in the dispersive media without the need to first filter the samples. In fact, in this study, the SPME devices were directly immersed in the dispersive media, while filtration was necessary before eluting the sample in the µSPE cartridges to avoid clogging. Both methods required less than 10 mL of sample and used the same quantitative analysis and instrumentation. Overall, both techniques exhibited good quantitative performance.

## Conclusion

This study investigated the extraction of PFAS compounds from mascara products in H_2_O/CH_3_OH media using both SPME and µSPE methods. Each technique provided distinct advantages and limitations. The results showed that using 100% H_2_O as a dispersive media improved the extraction efficiency for hydrophilic PFAS with SPME, while 100% CH_3_OH proved to be optimal for the removal of hydrophobic PFAS, such as PFOS, 8:2 FTS, and 6:2 diPAP, with µSPE. SPME enabled faster sample preparation by allowing direct immersion of the devices into the mascara mixture, made possible by the biocompatible PAN binder used with the WAX particles. In contrast, although µSPE was automated, it required pre-filtration of samples to prevent clogging and contamination of the PAL autosampler. SPME and µSPE showed a wide linear dynamic range (0.025–25 ng/g) and their performance demonstrated that µSPE achieved lower LOQs compared to SPME, especially for PFOS, 8:2 FTS, and 6:2 diPAP the most hydrophobic PFAS. PFOA was detected in six of the nine mascaras while 6:2 diPAP was quantified in four of the nine mascaras tested. Instrument, solvent, thin film, and cartridge blanks were monitored to ensure no PFAS interference and blank subtractions were performed where necessary. This study lays the foundation for the application of SPME and µSPE to a wide range of personal care products and cosmetics. Continued monitoring of PFAS in such products is crucial and strict regulations should be put in place to control their use, as direct human exposure is inevitable.

## Supplementary Information

Below is the link to the electronic supplementary material.

Supplementary file1 (PDF 1905 KB)

## Data Availability

All data supporting the findings of this study are available within the paper and its Supplementary Information.
