# Supplementary material for: Evaluation of extraction methodologies for PFAS analysis in mascara: a comparative study of SPME and automated µSPE
Source: Anal Bioanal Chem. 2025 May 24;418(2):619–32. doi: 10.1007/s00216-025-05908-x (PMC12783168; doi:10.1007/s00216-025-05908-x)
Supplement: Supplementary file 1 — Supplementary file1 (PDF 1905 KB) [file 216_2025_5908_MOESM1_ESM.pdf]

## Supplementary Information

# Evaluation of Extraction Methodologies for PFAS Analysis in Mascara: Comparative Study of SPME and Automated µSPE

*Aghogho A. Olomukoro [1], Lucas Lüthy [2], Tom Flug [2], Emanuela Gionfriddo [1, *]*

*[1]Department of Chemistry, University at Buffalo, The State University of New York, Buffalo, NY, 14260-3000, USA*

*[2]CTC Analytics AG Industriestrasse 20, 4222 Zwingen Switzerland*

*Corresponding author: egionfri@buffalo.edu

**Table of Content**


| Contents | Page number |
|---|---|
| Section 1 | S3 |
| Section 2 | S4 |
| Section 3 | S5 |
| Section 4 | S6 |
| Table S1 | S8 |
| Table S2 | S9 |
| Table S3 | S10 |
| Table S4 | S11 |
| Table S5 | S12 |
| Table S6 | S13 |
| Table S7 | S14 |
| Table S8 | S15 |
| Table S9 | S16 |
| Figure S1 | S17 |
| Figure S2 | S18 |
| Figure S3 | S18 |
| Figure S4 | S19 |
| Figure S5 | S20 |
| Figure S6 | S21 |
| Figure S7 | S23 |
| Reference | S24 |

**Section 1- Materials**

Perfluorobutanesulfonate (PFBS), PFHxA, PFHpA, PFOA, PFOS, 4:2 fluorotelomer sulfonic acid (4:2 FTS), 8:2 fluorotelomer sulfonic acid (8:2 FTS), 6:2 fluorotelomer phosphate diester (6:2 diPAP) were obtained from AccuStandard (New Haven, CT, USA) and Wellington Laboratories (Guelph, ON, Canada) and stored in the freezer or ambient conditions as recommended by the manufacturer and the details of these compounds can be found in **Table S1**. Isotopically labelled internal standards (PFHxA-C13, GenX-C13, PFOA-C13, PFOS-C13 and 6:2 diPAP-C13) were purchased from Wellington Laboratories (Guelph, ON, Canada). Ultrapure water used for the experiments was obtained by a water purification system Avidity Science, Alto I, Type 1, (San Diego, CA, USA) was used for all experiments and LC-MS grade methanol ($CH_3OH$) was purchased from Fisher Scientific (Hampton, NH, USA). 2 mL LC glass vials, 700 µL plastic vials and 0.22 µm nylon syringe filters with diameters of 4 mm and 13 mm were purchased from Microsolv (Leland, NC, USA). 500 µL plastic vials were obtained from Lab Depot INC (Dawsonville, GA, USA). Ammonium acetate and ammonium formate were purchased from Fisher Scientific (Hampton, NH, USA). All mascara products were purchased from Amazon, with manufacturers based in the USA, Italy, Luxembourg and Russia. The mascara products were named as product 1 (P1), product 1 waterproof (P1W), product 2 waterproof (P2W), product 3 (P3), product 4 (P4) and product 4 waterproof (P4W). PAL System µSPE C18 (CEC18 – 15 mg) and WAX (WAX Phenomenex - 50 mg) cartridges were provided to us by CTC analytics AG (Zwingen, Switzerland). SPME fibers and TF-SPME were prepared according to the procedure described in [1]. Polyacrylonitrile (PAN) were purchased from Sigma Aldrich (St. Loius, MO, USA).

**Section 2 - Preparation of the SPME fiber and thin film**

HLB-WAX particles (30 µm, Waters Corportaion, Milford, MA, USA) were mixed with PAN at a 9:1 mass ratio of PAN to particles to prepare HLB-WAX/PAN sorbents, which were immobilized on a stainless steel blade (Component Supply, Fort Meade, FL, USA) or nitinol wire (Yarder Manufacturing Co., Toledo, OH, USA) to create a SPME thin film/fiber device according to the procedure described in [1]. The thin films were cleaned by sonication in a solution of $CH_3OH$ and isopropanol (50:50, v:v) for 30 min, dried and etched with sandpaper. The etched thin films were then sonicated in methanol for 30 min and dried. The nitinol wires were etched by sonicating for 30 min in $CH_3OH$.

**Section 3 - Liquid chromatography and Mass Spectrometry conditions**

For method optimization, a Dionex UltiMate 3000 RS UHPLC system, comprising a pump, autosampler (loop size 25 µL), and column compartment (Thermo Fisher Scientific, Waltham, MA, USA), was used for chromatographic separation. The separation was performed on a Perkin Elmer Brownlee SPP C18 column (50 mm × 3 mm, 2.7 µm), with the column compartment maintained at 30 °C. Additionally, a PFAS delay column (Phenomenex Luna C18(2) 100 Å, 30 mm × 3 mm, 5 µm) was used to trap and delay instrument related PFAS. The total runtime was 10 min, with an injection volume of 10 µL. A mobile phase consisting of 2.5 mM ammonium acetate in ultrapure water (A) and methanol (B) was used in gradient mode. The flow rate was set at 0.5 mL/min, starting with 5% B from 0 to 1.0 min, increasing to 45% by 1.5 min, followed by 98% B from 7 to 8.0 min, and returning to 5% B from 8.1 to 10 min. Detection and quantification of analytes were carried out using a TSQ Endura triple quadrupole mass spectrometer (Thermo Fisher Scientific, Waltham, MA, USA) equipped with a heated electrospray ionization (HESI) source in negative mode. Selected Reaction Monitoring (SRM) was employed to monitor all target analytes and internal standards (**Table S2**). The optimized mass spectrometry conditions included an ESI voltage of -2500 V, with sheath, auxiliary, and sweep gases set at 40, 20, and 8 (arbitrary units), respectively. The vaporization and ion transfer tube temperatures were 350 °C and 250 °C, respectively.

For method validation, a QSight LX50 binary UHPLC system (PerkinElmer Inc., Waltham, MA, USA) with a pump, autosampler (partial loop injection, total loop size 20 µL), and column compartment was used for chromatographic separation, following the same chromatographic conditions as described above. Detection and quantification were performed using a QSight 220 triple quadrupole mass spectrometer (PerkinElmer Inc., Waltham, MA, USA) equipped with HESI

in negative mode. Multiple Reaction Monitoring (MRM) was applied to monitor all target analytes and internal standards (**Table S3**). Nitrogen gas, provided by a Peak Scientific Genius XE QSD nitrogen generator (Peak Scientific Instruments Ltd, Inchinnan, United Kingdom), was used for the ESI source, laminar flow ion guide, and collision cell. The optimized mass spectrometry conditions included an ESI voltage of -2300 V, with drying and nebulizer gases set to 100 and 200 (arbitrary units), respectively. The source and HSID temperatures were set at 250°C and 200°C, respectively.

**Section 4 - Automated PAL system procedure**

In this study, the automated PAL system was operated offline. However, it is equipped with various tools that allow it to perform functions both offline and online, such as calibration, dilution, µSPE pre-concentration and direct injection into LC or GC systems. A custom script is written to run a specific method, depending on the requirements of the work. The µSPE method includes the following steps:

1. The µSPE syringe is picked up from the parking station, mounted on the robotic arm and moved to the wash station.
2. The syringe draws the designated volume of conditioning solution and moves it to the cartridge tray for conditioning.
3. After conditioning, the syringe moves to the sample tray, draws the required volume of sample and loads it onto the cartridge at a rate of 1 to 30 µL/s, directing the flow into the waste.

4. Before elution, the syringe is washed and loaded with the elution solution. It then grips the cartridge and transfers it to the elution tray with empty vials. The elution solution can be passed through the cartridge at a flow rate range of 1 to 30 μL/s.
5. After elution, the cartridge is either discarded or returned to its position on the cartridge tray and the syringe is rinsed. The system then moves on to the next sample, or the syringe returns to the home position when all samples have been processed. A schematic diagram of the PAL autosampler is shown in **Fig. S1** below.

**Table S1:** Target analyte information

| Analytes | Abbreviations | Molecular weight (g/mol) | pKa [a] | Log P [a] |
|---|---|---|---|---|
| Perfluorobutane sulfonate | PFBS | 300.09 | -3.31 | 2.63 |
| Perfluorohexanoic acid | PFHxA | 314.054 | 0.32 | 3.71 |
| Perfluoroheptanoic acid | PFHpA | 364.062 | 0.31 | 4.41 |
| Perfluorooctanoic acid | PFOA | 414.07 | 0.3 | 5.11 |
| Perfluorooctane sulfonate | PFOS | 500.13 | -3.32 | 5.43 |
| 1H,1H,2H,2H perfluorohexanesulfonic acid | 4:2 FTS | 328.15 | -2.64 | 2.52 |
| 1H,1H,2H,2H perfluorodecanesulfonic acid | 8:2 FTS | 528.18 | -2.61 | 5.32 |
| Bis(1H,1H,2H,2Hperfluorooctyl) phosphate | 6:2 diPAP | 790.17 | 1.94 | 9.19 |

[a] pKa and Log P – Chemicalize, Chemaxon https://chemicalize.com/app/calculation

**Table S2**: Optimized MS/MS Parameters for PFAS Analysis on the TSQ Endura

| Compound | Precursor (m/z) | Product (m/z) | Type | Collision Energy (V) | RF Lens (V) |
|---|---|---|---|---|---|
| PFBS | 298.9 | 79.8 | Quantifier | 32.9 | 156 |
| PFBS | 298.9 | 98.7 | Qualifier | 28.5 | 156 |
| PFHxA | 312.8 | 118.8 | Qualifier | 18.5 | 64 |
| PFHxA | 312.8 | 268.8 | Quantifier | 10.2 | 64 |
| PFHpA | 362.8 | 168.9 | Qualifier | 15.1 | 75 |
| PFHpA | 362.8 | 318.8 | Quantifier | 10.2 | 75 |
| PFOA | 412.8 | 168.8 | Qualifier | 16.1 | 83 |
| PFOA | 412.8 | 368.8 | Quantifier | 10.2 | 83 |
| PFOS | 498.8 | 79.8 | Quantifier | 44.1 | 178 |
| PFOS | 498.8 | 98.8 | Qualifier | 38.1 | 178 |
| 4:2 FTS | 326.8 | 80.8 | Quantifier | 26.5 | 133 |
| 4:2 FTS | 326.8 | 286.9 | Qualifier | 22.6 | 133 |
| 4:2 FTS | 326.8 | 306.9 | Qualifier | 17.7 | 133 |
| 8:2 FTS | 526.8 | 80.7 | Quantifier | 31.5 | 165 |
| 8:2 FTS | 526.8 | 506.9 | Qualifier | 24.9 | 165 |
| 6:2 diPAP | 788.9 | 96.9 | Quantifier | 27.4 | 299 |
| 6:2 diPAP | 788.9 | 422.8 | Qualifier | 22.7 | 299 |

**Table S3:** Optimized MS/MS Parameters for PFAS Analysis on the QSight 220

| Name | Q1 mass | Q2 mass | Type | Internal Standard | CE | EV | CCL2 |
|---|---|---|---|---|---|---|---|
| PFBS | 299.1 | 80 | Quantifier | $^{13}C_3$ GenX | 67 | -37 | 80 |
| PFBS | 299.1 | 99 | Qualifier | $^{13}C_3$ GenX | 39 | -43 | 56 |
| PFHxA | 312.9 | 268.9 | Quantifier | $^{13}C_2$ PFHxA | 14 | -2 | 56 |
| PFHxA | 312.9 | | Qualifier | $^{13}C_2$ PFHxA | | | |
| PFHpA | 362.9 | 168.9 | Qualifier | $^{13}C_3$ GenX | 23 | -9 | 72 |
| PFHpA | 362.9 | 318.9 | Quantifier | $^{13}C_3$ GenX | 14 | -11 | 72 |
| PFOA | 412.9 | 168.9 | Qualifier | $^{13}C_8$ PFOA | 26 | 0 | 76 |
| PFOA | 412.9 | 368.9 | Quantifier | $^{13}C_8$ PFOA | 13 | -2 | 80 |
| PFOS | 498.9 | 80 | Quantifier | $^{13}C_8$ PFOS | 90 | -50 | 140 |
| PFOS | 498.9 | 99 | Qualifier | $^{13}C_8$ PFOS | 55 | -40 | 130 |
| 4:2 FTS | 326.9 | 81 | Quantifier | $^{13}C_3$ GenX | 51 | 0 | 68 |
| 4:2 FTS | 326.9 | 287 | Qualifier | $^{13}C_3$ GenX | 31 | -2 | 100 |
| 4:2 FTS | 326.9 | 306.9 | Qualifier | $^{13}C_3$ GenX | 26 | -2 | 68 |
| 8:2 FTS | 527.1 | 81 | Quantifier | $^{13}C_8$ PFOS | 91 | -20 | 148 |
| 8:2 FTS | 527.1 | 306.9 | Qualifier | $^{13}C_8$ PFOS | 37 | -14 | 164 |
| 6:2 diPAP | 789 | 97 | Quantifier | | 48 | -44 | 140 |
| 6:2 diPAP | 789 | 422.8 | Qualifier | | 35 | -43 | 140 |
| $^{13}C_3$ GenX | 287 | 184.9 | Internal standard | - | 27 | -10 | 56 |
| $^{13}C_2$ PFHxA | 315 | 269.9 | Internal standard | - | 12 | -8 | 64 |
| $^{13}C_8$ PFOA | 421 | 375.9 | Internal standard | - | 14 | -7 | 80 |
| $^{13}C_8$ PFOS | 507.3 | 79.9 | Internal standard | - | 99 | -60 | 140 |
| $^{13}C_2$ 6:2 diPAP | 793 | 444.9 | Internal standard | - | 26 | -26 | 168 |

**Table S4**: µSPE matrix effect calculated for the different mascara products

| | **µSPE Matrix effect (%)** | | | | | |
|---|---|---|---|---|---|---|
| | Product 1 | Product 1 Waterproof | Product 2 | Product 3 | Product 4 | Product 4 Waterproof |
| **PFBS** | 73.9 ± 9.6 | 93.6 ± 13.8 | 86.7 ± 6.1 | 83.2 ± 5.3 | 87.0 ± 7.7 | 82.2 ± 14.1 |
| **4:2 FTS** | 92.7 ± 8.2 | 86.3 ± 13.0 | 76.8 ± 5.2 | 79.9 ± 7.8 | 81.8 ± 9.4 | 74.5 ± 11.6 |
| **PFHxA** | 72.7 ± 11.0 | 88.1 ± 12.6 | 80.0 ± 6.1 | 84.2 ± 7.3 | 86.5 ± 9.0 | 76.1 ± 13.2 |
| **PFHpA** | 67.4 ± 9.8 | 83.1 ± 11.8 | 81.6 ± 4.0 | 100.3 ± 9.5 | 95.1 ± 11.1 | 76.8 ± 13.1 |
| **PFOA** | 78.8 ± 9.9 | 90.6 ± 13.6 | 110.5 ± 7.7 | 89.8 ± 8.5 | 110.9 ± 10.9 | 80.8 ± 13.6 |
| **PFOS** | 80.3 ± 11.5 | 91.7 ± 12.6 | 84.6 ± 9.0 | 92.6 ± 8.1 | 93.8 ± 10.7 | 81.0 ± 11.7 |
| **8:2 FTS** | 113.9 ± 17.1 | 109.8 ± 18.9 | 95.9 ± 5.8 | 102.0 ± 6.7 | 282.0 ± 35.0 | 100.9 ± 17.4 |
| **6:2 diPAP** | 383.3 ± 62.6 | 176.7 ± 15.0 | 166.0 ± 11.7 | 147.5 ± 12.4 | 336.4 ± 52.1 | 93.4 ± 17.4 |

Number of replicates n=6 (± standard error)

**Table S5**: μSPE matrix effect calculated for P4W with the inclusion of a washing step and internal standards

| | Matrix effect (%) | | | |
|---|---|---|---|---|
| | Washing method | Washing method with ISTD | No washing method | No washing method with ISTD |
| **PFBS** | 74.5 ± 2.4 | 103.3 ± 16.3 | 83.5 ± 5.3 | 104.0 ± 13.3 |
| **4:2 FTS** | 73.9 ± 1.7 | 103.7 ± 16.2 | 84.7 ± 5.5 | 101.8 ± 13.3 |
| **PFHxA** | 71.0 ± 1.5 | 97.0 ± 15.3 | 79.1 ± 4.5 | 97.9 ± 12.4 |
| **PFHpA** | 71.7 ± 3.2 | 102.6 ± 14.2 | 80.8 ± 4.2 | 95.2 ± 3.0 |
| **PFOA** | 82.9 ± 12.3 | 106.2 ± 14.4 | 86.6 ± 4.5 | 95.4 ± 2.6 |
| **PFOS** | 70.8 ± 3.4 | 103.1 ± 17.6 | 82.6 ± 5.3 | 80.5 ± 16.8 |
| **8:2 FTS** | 80.6 ± 10.6 | 110.5 ± 18.3 | 119.4 ± 19.7 | 99.8 ± 21.4 |
| **6:2 diPAP** | 26.5 ± 4.8 | 99.2 ± 12.7 | 167.7 ± 20.2 | 82.8 ± 5.8 |

Number of replicates n=6 (± standard error)

**Table S6:** Accuracy and precision for µSPE protocol: Day 1, Day 5 and Day 7

| | | | | | | | | |
|---|---|---|---|---|---|---|---|---|
| | | | | **Day 1** | | | | |
| **Concentrations** | PFBS | 4:2 FTS | PFHxA | PFHpA | PFOA | PFOS | 8:2 FTS | 6:2 diPAP |
| **0.25 ng/g** | 81.0 % (12.4 %) | 95.2 % (11.5 %) | 110.7 % (8.1 %) | 120.0 % (7.2 %) | 110.8 % (9.5 %) | 114.4 % (8.5 %) | 107.4 % (3.6 %) | -* |
| **0.75 ng/g** | 124.0 % (5.7 %) | 120.2 % (13.5%) | 121.0 % (5.1 %) | 115.0 % (4.8 %) | 114.9 % (2.4 %) | 99.0 % (5.0 %) | 101.5 % (8.9 %) | 128.9 % (1.5 %) |
| **2 ng/g** | 111.7 % (5.4 %) | 107.5 % (8.6 %) | 106.7 % (4.5 %) | 100.6 % (2.7 %) | 102.4 % (2.2 %) | 83.0 % (8.5 %) | 110.2 % (1.1 %) | 97.4 (4.6 %) |
| **7.5 ng/g** | 119.6 % (3.5 %) | 116.1 % (4.3 %) | 114.6 % (3.3 %) | 113.6 % (1.7 %) | 109.6 % (1.3 %) | 97.5 % (5.5 %) | 99.8 % (1.1 %) | 81.7 (2.9 %) |
| | | | | **Day 5** | | | | |
| **0.25 ng/g** | 110.9 % (8.4 %) | 116.3 % (10.8 %) | 123.0 % (4.7 %) | 159.7 % (13.1 %) | 132.2 % (4.5 %) | 145.3 % (13.1 %) | 14.7 % (15.0 %) | -* |
| **0.75 ng/g** | 133.0 % (7.2 %) | 133.7 % (3.6 %) | 130.4 % (13.2 %) | 165. 5 % (11.0 %) | 134.7% (2.7 %) | 131.0 % (2.5 %) | 217.6 % (11.6 %) | -* |
| **2 ng/g** | 112.7 (6.8 %) | 123.6 (6.0 %) | 115.3 % (4.4 %) | 136.6 % (4.2 %) | 114.7 % (3.9 %) | 113.0 % (6.3 %) | 166.6 % (6.6 %) | -* |
| **7.5 ng/g** | 123.3 (5.1 %) | 129.7 (1.5 %) | 124.6 % (3.1 %) | 145.4 % (5.0 %) | 124.5 % (4.1 %) | 113.7 % (5.6 %) | -* | -* |
| | | | | **Day 7** | | | | |
| **0.25 ng/g** | 101.7 % (12.1 %) | 74.0 % (13.0 %) | 107.8 % (11.6 %) | 123.1 % (8.2 %) | 125.5 % (7.2 %) | 114.0 % (9.7 %) | 50.2 % (10.2 %) | -* |
| **0.75 ng/g** | 121.9 % (6.5 %) | 91.6 % (8.0 %) | 120.2 % (6.5 %) | 121.1 % (4.5 %) | 130.1 % (3.1 %) | 103.4 % (5.7 %) | 102.7 % (8.6 %) | 116.0 % (5.9 %) |
| **2 ng/g** | 112.7 (5.6 %) | 123.6 % (6.8 %) | 115.3 % (5.2 %) | 136.6 % (3.3 %) | 114.7 % (3.8 %) | 88.3 % (4.4 %) | 102.1 % (7.7 %) | 89.6 % (11.7 %) |
| **7.5 ng/g** | 111.2 (4.7 %) | 91.0 % (5.0 %) | 108.7 % (1.6 %) | 108.6 % (1.6 %) | 111.8 % (1.1 %) | 84.2 % (2.3 %) | 105.3 % (14.7 %) | 96.7 % (8.6 %) |

*not within the linear range

**Table S7:** Accuracy and precision for SPME protocol: Day 1, Day 5 and Day 7

| Concentrations | PFBS | 4:2 FTS | PFHxA | PFHpA | PFOA | PFOS | 8:2 FTS | 6:2 diPAP |
|---|---|---|---|---|---|---|---|---|
| **Day 1** | | | | | | | | |
| **0.25 ng/g** | 94.0 % (17.6 %) | 99.6 % (10.2 %) | 96.1 % (15.5 %) | 60.3 % (6.5 %) | 87.1 % (7.5 %) | 96.1 % (15.5 %) | -* | -* |
| **0.75 ng/g** | 98.8 % (9.7 %) | 119.6 % (3.3 %) | 116.0 % (12.4 %) | 130.0 % (4.4 %) | 112.2 % (7.7 %) | 116.0 % (12. 4%) | 101.3 % (8.8 %) | -* |
| **2 ng/g** | 78.5 % (10.5 %) | 103.8 % (2.4 %) | 100.2 % (2.3 %) | 127.2 % (5.3 %) | 96.4 % (3.4 %) | 100.3 % (2.3 %) | 92.3 % (11.0 %) | 55.9 % (4.4 %) |
| **7.5 ng/g** | 93.0 % (5.0 %) | 100.7 % (3.7 %) | 99.0 % (1.8 %) | 122.0 % (8.4 %) | 94.0 % (1.8 %) | 99.0 % (1.8 %) | 99.7 % (20.1 %) | 73.5 % (0.5 %) |
| **Day 5** | | | | | | | | |
| **0.25 ng/g** | 109.9 % (15.5 %) | 101.0 % (15.0 %) | 98.5 % (15.2 %) | 113.1 % (4.4 %) | 99.1 % (7.1 %) | 101.3 % (15.0 %) | -* | -* |
| **0.75 ng/g** | 98.8 % (14.8 %) | 122.7 % (16.7 %) | 105.9 % (9.8 %) | 73.1 % (6.5 %) | 99.8 % (9.7 %) | 105.9 % (9.8 %) | 116.2 % (8.5 %) | -* |
| **2 ng/g** | 77.6 % (6.7 %) | 106.8 % (5.3 %) | 93.6 % (1.0 %) | 94.2 % (6.3 %) | 85.7 % (1.2 %) | 93.7 % (1.0 %) | 186.1 % (7.2 %) | 573.4 % (1.0 %) |
| **7.5 ng/g** | 85.8 % (5.5 %) | 93.7 % (2.6 %) | 92.0 % (1.1 %) | 85.0 % (8.4 %) | 82.7 % (1.2 %) | 92.0 % (1.1%) | 153.8 % (2.0 %) | 558.0 % (1.1 %) |
| **Day 7** | | | | | | | | |
| **0.25 ng/g** | 109.7 % (11.6 %) | 154.1 % (7.6 %) | 97.4 % (18.5 %) | 132.4 % (4.8 %) | -* | -* | -* | **-*** |
| **0.75 ng/g** | 107.4 % (3.2 %) | 188.4 % (4.2 %) | 104.8 % (11.6 %) | 71.4 % (11.3 %) | 96.3 % (6.7 %) | 83.5 % (13.8 %) | -* | -* |
| **2 ng/g** | 73.0 % (17.6 %) | 170.3 % (11.9 %) | 90.8 % (1.3 %) | 93.2 % (8.6 %) | 83.5 (6.6 %) | 126.3 % (17.3 %) | -* | 23.7 % (5.6 %) |
| **7.5 ng/g** | 70.1 % (5.7 %) | 150.0 % (3.1 %) | 95.4 % (3.3 %) | 86.2 % (3.0%) | 86.2 % (1.5 %) | 131.7 % (7.0) % | -* | 10.3 % (16.8 %) |

*not within the linear range

**Table S8:** Analysis of real samples using µSPE method

| Analytes | P1 USA | P1W USA | P2W Luxembourg | P3 USA | P4 USA | P4W USA | P5 Italy | P6 Russia | P7 Italy |
|---|---|---|---|---|---|---|---|---|---|
| PFBS | x | x | x | x | x | x | x | x | x |
| 4:2 FTS | x | x | x | x | x | x | x | x | x |
| PFHxA | x | x | x | x | x | x | x | x | x |
| PFHpA | x | x | x | x | x | x | x | x | x |
| PFOA | x | x | x | x | x | x | x | x | <LOQ |
| PFOS | x | x | x | x | x | x | x | x | x |
| 8:2 FTS | x | x | x | x | x | x | x | x | x |
| 6:2 diPAP | 1.26 ± 0.19 ng/g | x | 3.48 ± 0.44 ng/g | x | x | 1.36 ± 0.17 ng/g | x | x | <LOQ |

* x indicates PFAS not detected

**Table S9**: Analysis of real samples using SPME method

| Analytes | P1<br>USA | P1W<br>USA | P2W<br>Luxembourg | P3<br>USA | P4<br>USA | P4W<br>USA | P5<br>Italy | P6<br>Russia | P7<br>Italy |
|---|---|---|---|---|---|---|---|---|---|
| PFBS | x | x | x | x | x | x | x | x | x |
| 4:2 FTS | x | x | x | x | x | x | x | x | x |
| PFHxA | x | x | x | x | x | x | x | x | x |
| PFHpA | x | x | x | x | x | x | x | x | x |
| PFOA | <LOQ | x | 3.04 ± 0.33 ng/g | <LOQ | x | <LOQ | <LOQ | x | <LOQ |
| PFOS | x | x | x | x | x | x | x | x | x |
| 8:2 FTS | x | x | x | x | x | x | x | x | x |
| 6:2 diPAP | 2.21 ± 0.19 ng/g | x | 2.14 ± 0.29 ng/g | x | x | 1.38 ± 0.19 ng/g | x | x | <LOQ |

* x indicates PFAS not detected

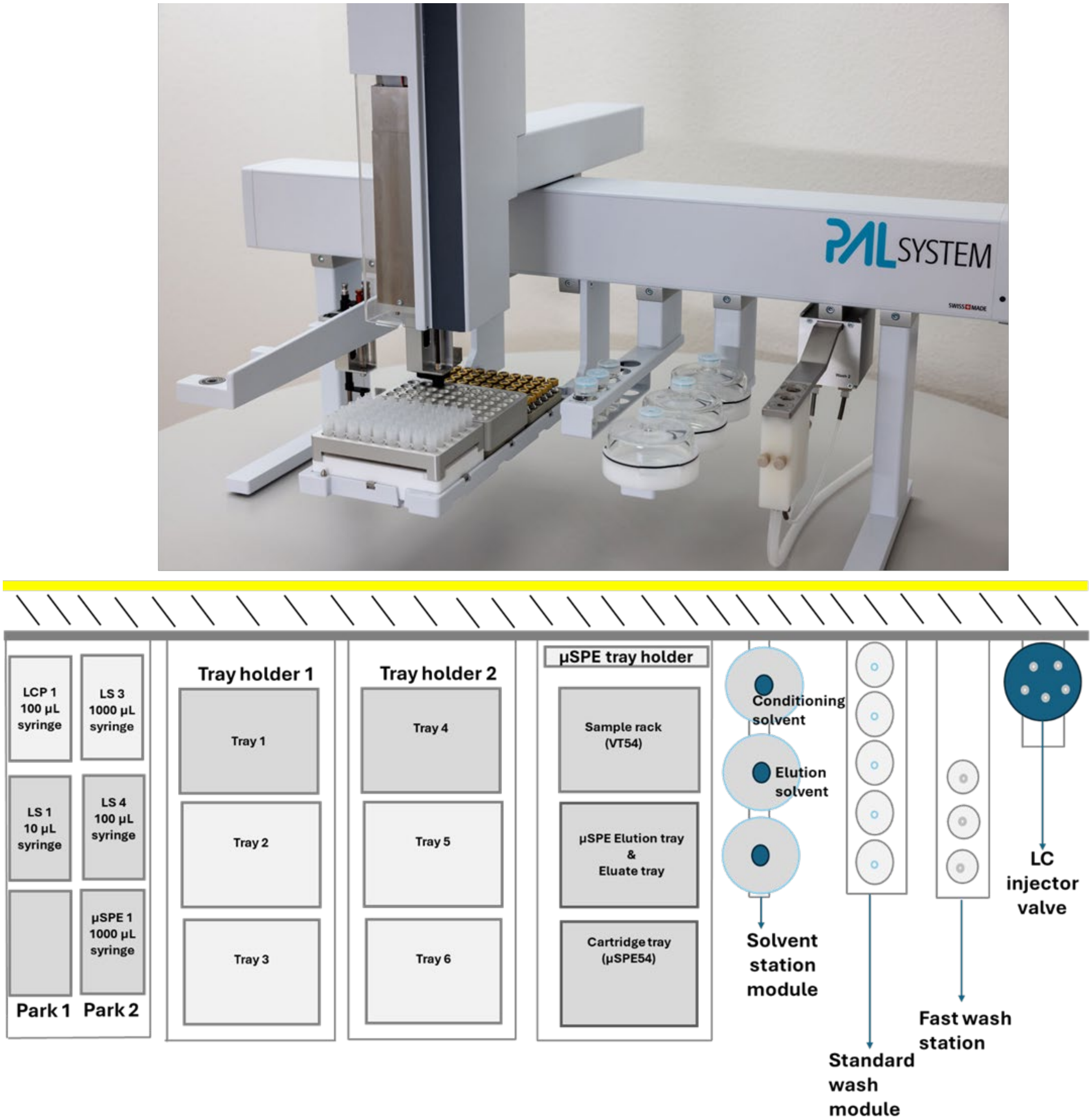


**Fig. S1** Schematic of the PAL autosampler for automated µSPE

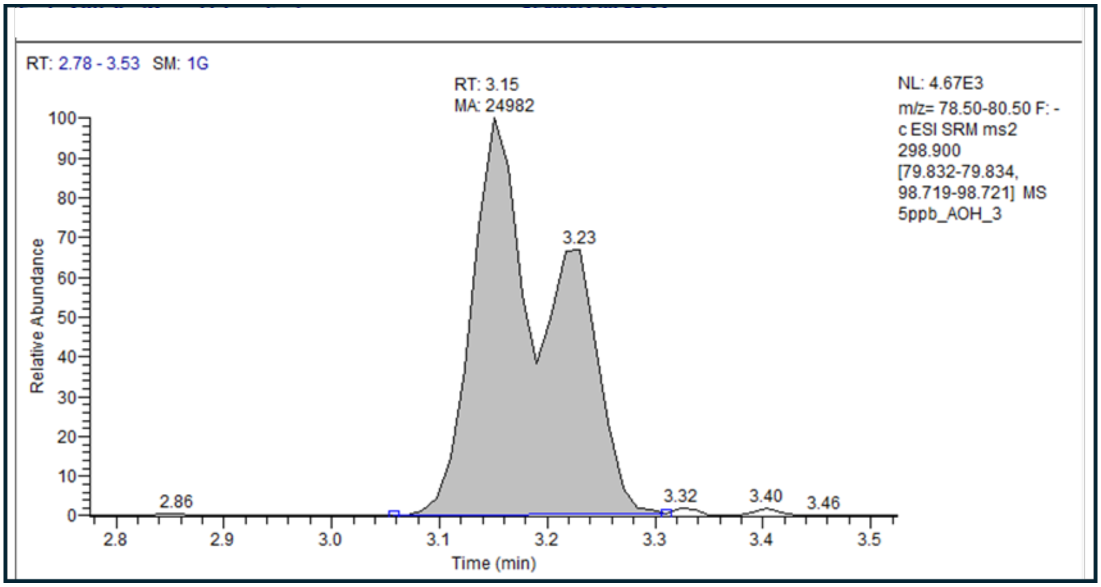


**Fig. S2** Chromatogram showing a distorted peak for PFBS when injected in 100% $CH_3OH$ with 0.5 % ammonium hydroxide as an additive

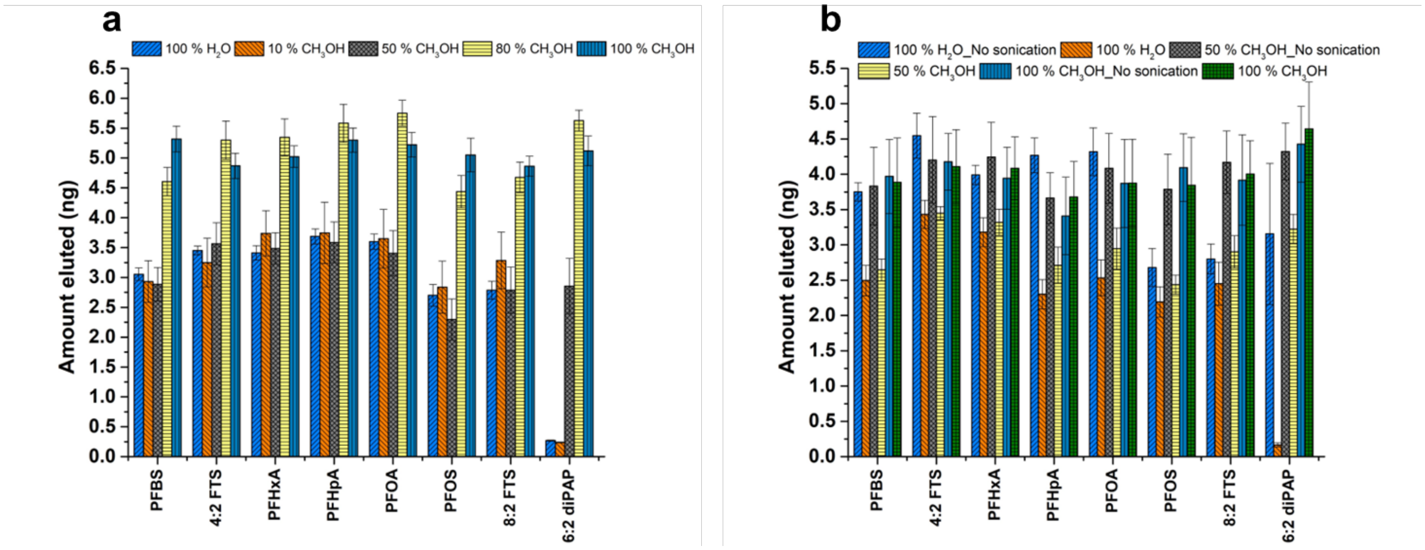


**Fig. S3** Loading solution composition, a) Sonication in 100 % water, 90 % water/10 % $CH_3OH$, 50 % water/50 % $CH_3OH$, 20 % water/80 % $CH_3OH$ and 100 % $CH_3OH$, b) comparing sonication and no sonication in 100 % water, 50 % water/50 % $CH_3OH$ and 100 % $CH_3OH$

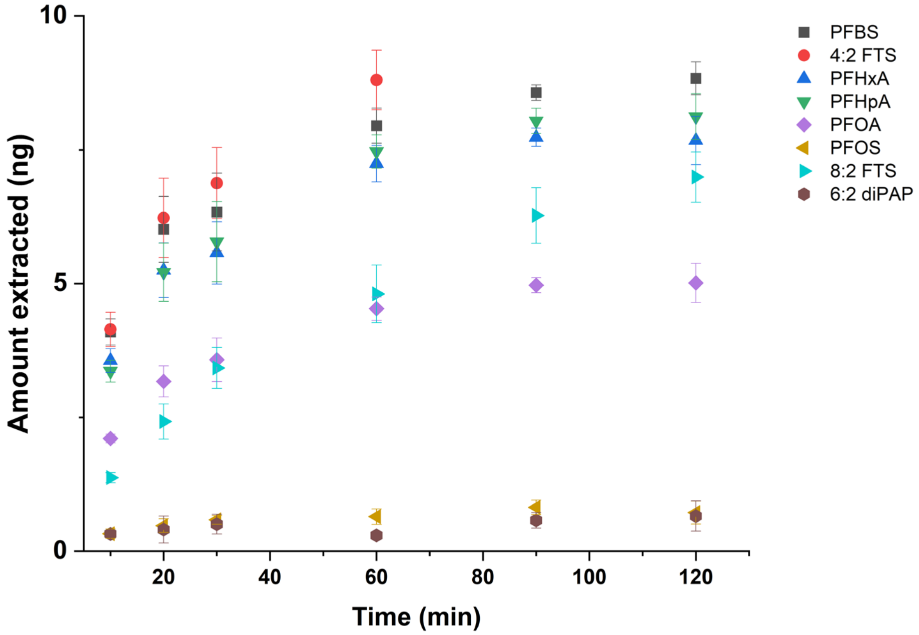


**Fig. S4** Extraction time profile performed at times 10, 20, 30, 60, 90 and 120 min using SPME thin film. Desorption was performed for 20 min in 80:20 $CH_3OH$:$H_2O$ (v:v) with 2 % ammonium formate. The agitation speed for extraction and desorption was 1000 rpm

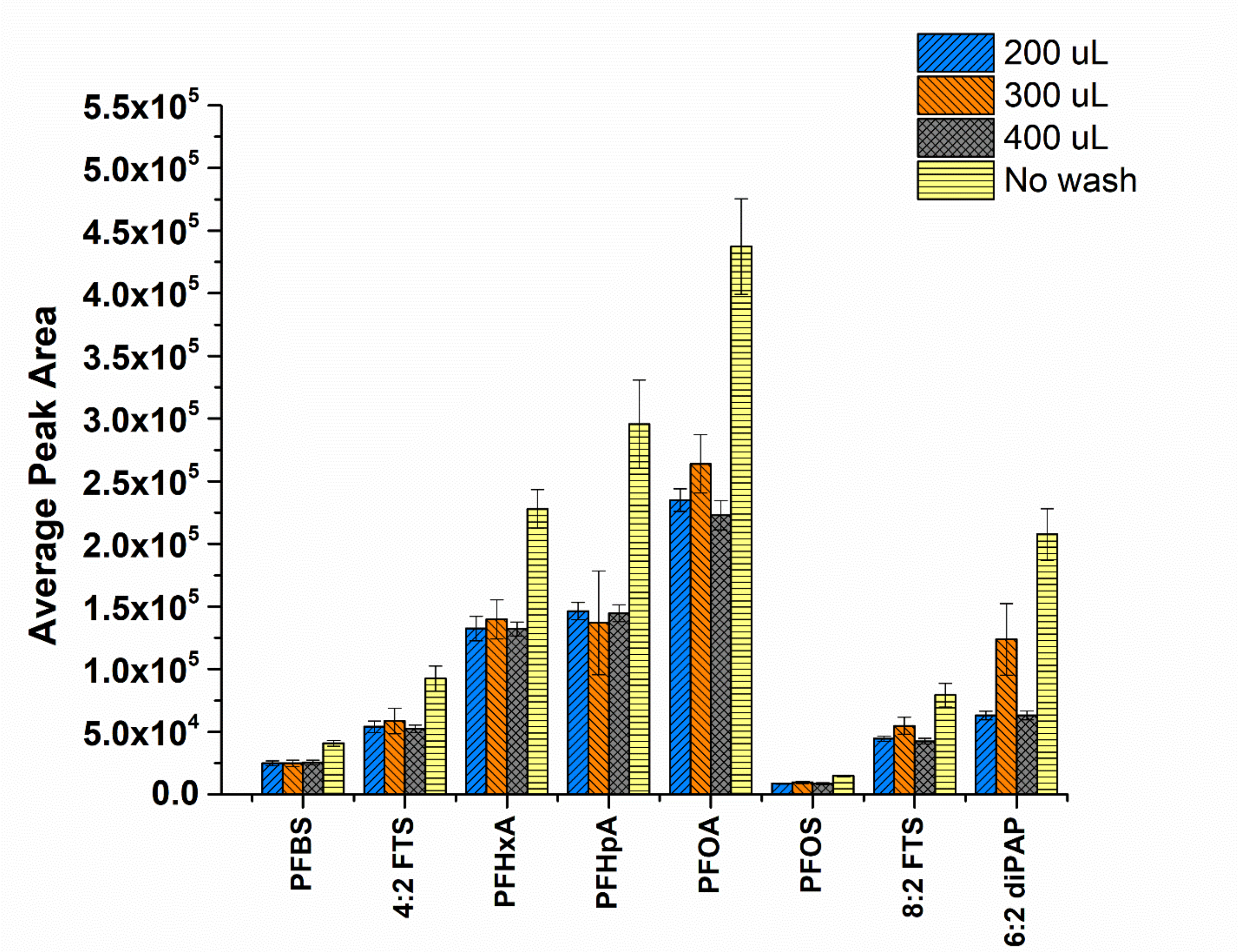


**Fig. S5** Washing step volume optimization. Sample and elution volume were 400 µL

a

# AGREEprep

## Analytical Greenness Metric for Sample Preparation

22/01/2025 20:40:50

Report

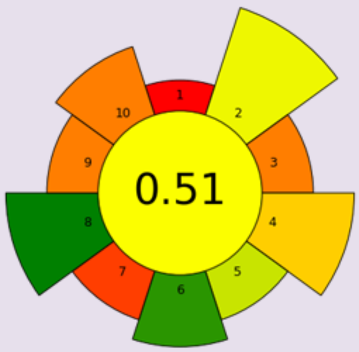


| # | Criterion | | Score | Weight |
|---|---|---|---|---|
| 1. | Sample preparation placement: | Ex situ | 0.00 | 1 |
| 2. | Hazardous materials: | 0.25 [g or mL] | 0.53 | 5 |
| 3. | Sustainability, renewability, and reusability of materials: | 25-50% of reagents and materials are sustainable or renewable | 0.25 | 2 |
| 4. | Waste: | 4 [g or mL] | 0.41 | 4 |
| 5. | Size economy of the sample | Mass or volume of the sample: 1.5 [g or mL] | 0.61 | 2 |
| 6. | Sample throughput: | 50 [samples/h] | 0.92 | 3 |
| 7. | Integration and automation | Sample prep. steps: 5 steps, Semi-automated systems | 0.13 | 2 |
| 8. | Energy consumption: | 1 [W] | 1.00 | 4 |
| 9. | Post-sample preparation configuration for analysis: | Liquid chromatography, gas chromatography with quadrupole detection, etc. | 0.25 | 2 |
| 10. | Operator's safety: | 3 hazards | 0.25 | 3 |

**b** AGREEprep

Analytical Greenness Metric for Sample Preparation

22/01/2025 20:48:58

Report

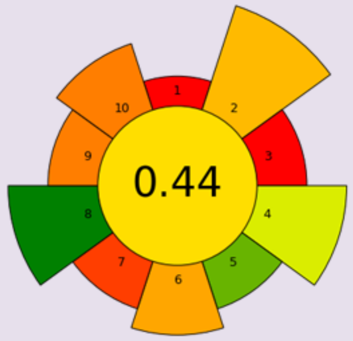


| # | Criterion | | Score | Weight |
|---|---|---|---|---|
| 1. | Sample preparation placement: | Ex situ | 0.00 | 1 |
| 2. | Hazardous materials: | 0.8 [g or mL] | 0.37 | 5 |
| 3. | Sustainability, renewability, and reusability of materials: | < 25% of reagents and materials are sustainable or renewable, but can only be used ONCE | 0.00 | 2 |
| 4. | Waste: | 1.4 [g or mL] | 0.58 | 4 |
| 5. | Size economy of the sample | Mass or volume of the sample: 0.4 [g or mL] | 0.80 | 2 |
| 6. | Sample throughput: | 4 [samples/h] | 0.33 | 3 |
| 7. | Integration and automation | Sample prep. steps: 5 steps, Semi-automated systems | 0.13 | 2 |
| 8. | Energy consumption: | 1 [W] | 1.00 | 4 |
| 9. | Post-sample preparation configuration for analysis: | Liquid chromatography, gas chromatography with quadrupole detection, etc. | 0.25 | 2 |
| 10. | Operator's safety: | 3 hazards | 0.25 | 3 |

**Fig. S6** AGREEprep metric assessment tool for a) SPME sample preparation, b) µSPE sample preparation

a

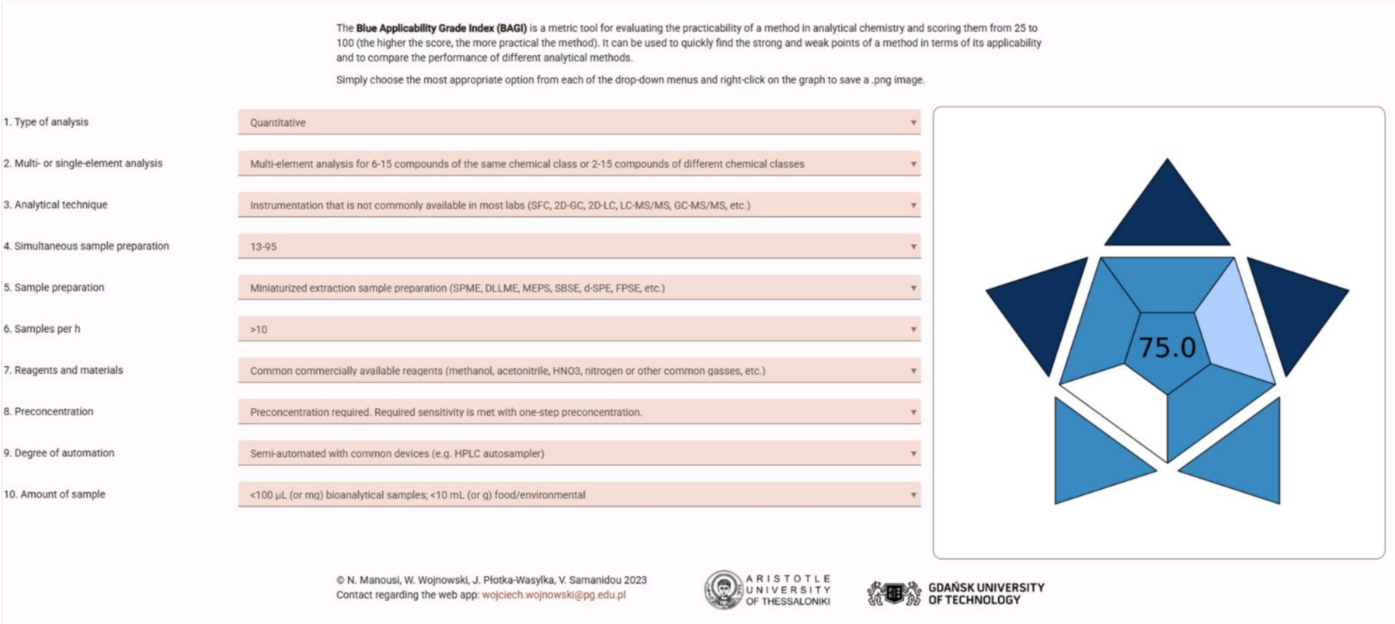


b

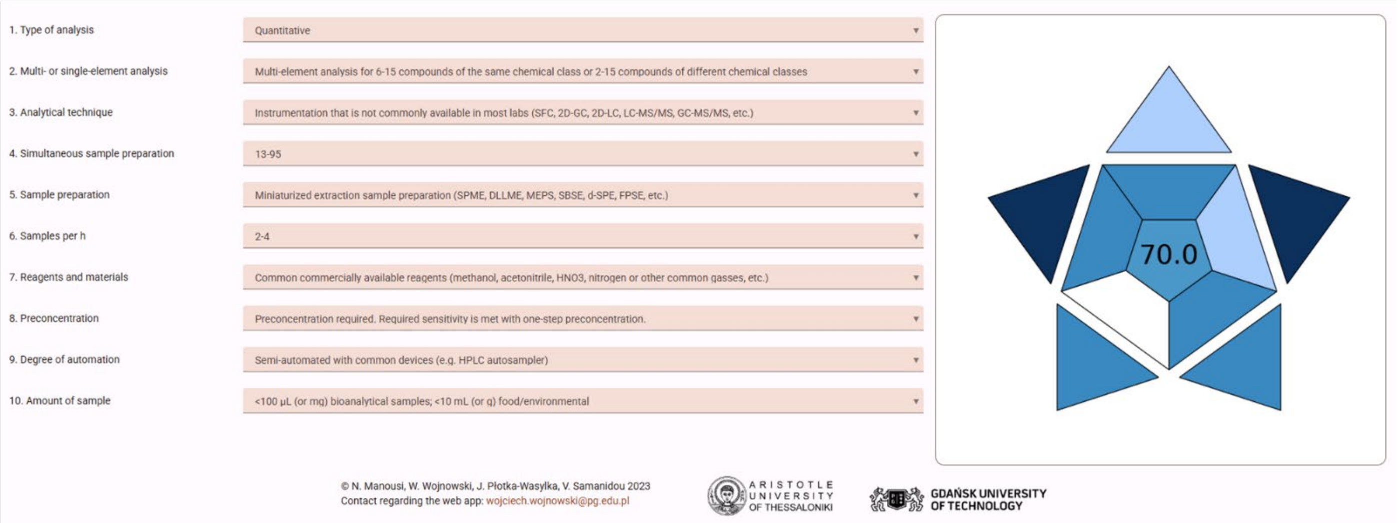


**Fig. S7** BAGI metric assessment tool for a) SPME sample preparation, b) µSPE sample preparation

## References


1. Olomukoro AA, DeRosa C, Gionfriddo E (2023) Investigation of the adsorption/desorption mechanism of perfluoroalkyl substances on HLB-WAX extraction phases for microextraction. Anal Chim Acta 1260:341206. https://doi.org/10.1016/j.aca.2023.341206
2. EPA (2018) EPA Method 537.1. 1:1–50
3. US EPA 8327 PER- AND POLYFLUOROALKYL SUBSTANCES (PFAS) BY LIQUID CHROMATOGRAPHY/TANDEM MASS SPECTROMETRY (LC/MS/MS)
4. Wendelken SC, Epa US (2018) Method 533:Determination of per-and polyfluoroalkyl substances in drinking water by isotope dilution snion exchange solid pahse extraction and liquid chromatography/tandem mass spectrometry 533-i,.
5. Matuszewski BK, Constanzer ML, Chavez-Eng CM (2003) Strategies for the assessment of matrix effect in quantitative bioanalytical methods based on HPLC-MS/MS. Anal Chem 75:3019–3030. https://doi.org/10.1021/ac020361s
6. Williams ML, Olomukoro AA, Emmons R V., Godage NH, Gionfriddo E (2023) Matrix effects demystified: Strategies for resolving challenges in analytical separations of complex samples. J Sep Sci 46
7. Gionfriddo E (2020) Biocompatible Microextraction Devices for Simple and Green Analysis of Complex Systems. LCGC North America 38:Number s6
